# Acute Overactive Endocannabinoid Signaling Induces Glucose Intolerance, Hepatic Steatosis, and Novel Cannabinoid Receptor 1 Responsive Genes

**DOI:** 10.1371/journal.pone.0026415

**Published:** 2011-11-04

**Authors:** Maxwell A. Ruby, Daniel K. Nomura, Carolyn S. S. Hudak, Anne Barber, John E. Casida, Ronald M. Krauss

**Affiliations:** 1 Department of Atherosclerosis Research, Children's Hospital Oakland Research Institute, Oakland, California, United States of America; 2 Department of Nutritional Sciences and Toxicology, University of California, Berkeley, California, United States of America; University of Minnesota, United States of America

## Abstract

Endocannabinoids regulate energy balance and lipid metabolism by stimulating the cannabinoid receptor type 1 (CB1). Genetic deletion and pharmacological antagonism have shown that CB1 signaling is necessary for the development of obesity and related metabolic disturbances. However, the sufficiency of endogenously produced endocannabinoids to cause hepatic lipid accumulation and insulin resistance, independent of food intake, has not been demonstrated. Here, we show that a single administration of isopropyl dodecylfluorophosphonate (IDFP), perhaps the most potent pharmacological inhibitor of endocannabinoid degradation, increases hepatic triglycerides (TG) and induces insulin resistance in mice. These effects involve increased CB1 signaling, as they are mitigated by pre-administration of a CB1 antagonist (AM251) and in CB1 knockout mice. Despite the strong physiological effects of CB1 on hepatic lipid and glucose metabolism, little is known about the downstream targets responsible for these effects. To elucidate transcriptional targets of CB1 signaling, we performed microarrays on hepatic RNA isolated from DMSO (control), IDFP and AM251/IDFP-treated mice. The gene for the secreted glycoprotein lipocalin 2 (lcn2), which has been implicated in obesity and insulin resistance, was among those most responsive to alterations in CB1 signaling. The expression pattern of IDFP mice segregated from DMSO mice in hierarchal cluster analysis and AM251 pre-administration reduced (>50%) the majority (303 of 533) of the IDFP induced alterations. Pathway analysis revealed that IDFP altered expression of genes involved in lipid, fatty acid and steroid metabolism, the acute phase response, and amino acid metabolism in a CB1-dependent manner. PCR confirmed array results of key target genes in multiple independent experiments. Overall, we show that acute IDFP treatment induces hepatic TG accumulation and insulin resistance, at least in part through the CB1 receptor, and identify novel cannabinoid responsive genes.

## Introduction

Obesity elicits a cluster of interrelated disorders, termed the “metabolic syndrome”, that increase the risk of cardiovascular disease [1]. Dysregulation of the endocannabinoid (EC) system has been linked to increased adiposity in humans by epidemiological and genetic data [2], [3], [4]. Obesity and hyperglycemia are associated with elevated plasma and tissue endocannabinoid levels in animal models and obese patients [2], [5], [6], [7]. In four large human trials, 20 mg/day of the cannabinoid type 1 receptor (CB1) antagonist rimonabant resulted in clinically significant and prolonged reductions in body weight, waist circumference, and components of the metabolic syndrome [8], [9], [10], [11]. The effects of rimonabant on plasma lipids and glycosylated hemoglobin appear to be partly independent of weight loss [12]. Pharmacological or genetic ablation of CB1 in diet-induced and genetic mouse models of obesity results in a transient hypophagic response, followed by prolonged effects on weight loss, adiposity, and normalization of metabolic parameters [13], [14], [15], [16], [17], [18]. These effects suggest that reduced food intake does not fully explain the improvement in adiposity-related measures with CB1 inactivation. Hepatic CB1 activation increases *de novo* lipogenesis through SREBP1c activation, and decreases fatty acid oxidation by inhibiting AMP kinase [19], [20]. Furthermore, hepatocyte specific deletion of CB1 or administration of a non-brain-penetrant CB1 antagonist prevents hepatic steatosis, hyperlipidemia, and insulin resistance on a high-fat diet, independent of weight gain [21], [22]. Similarly, ethanol-induced hepatic steatosis is absent in hepatocyte specific CB1 −/− animals [20]. Together these observations raise the possibility that aberrant EC signaling mediates development of the metabolic syndrome, both by influencing body weight and directly regulating metabolic processes.

While the necessity of CB1 signaling for development of obesity and related metabolic disturbances has been demonstrated, it is uncertain if EC elevation is sufficient to induce changes in hepatic lipid and glucose metabolism independent of changes in food intake and body weight. Furthermore, the molecular pathways underlying the powerful regulatory effects of CB1 on hepatic metabolism remain largely unclear. In the present study, we investigate the effects of elevated ECs on hepatic lipid content and insulin sensitivity independent of food intake. The ECs responsible for CB1 signaling are N-arachidonyl ethanolamine (AEA, or anandamide) and 2-arachidonoyl glycerol (2-AG). Both are arachidonic acid derivatives produced locally by phospholipases, N-acyl-phosphatidylethanolamine-selective phospholipase D, and sn-1-selective diacylglycerol lipases, respectively [23]. Anandamide is a partial CB1 agonist with moderate affinity, and 2-AG is a lower affinity complete CB1 agonist that is present at much higher concentrations than AEA. Signaling is terminated by enzymatic breakdown of AEA and 2-AG by fatty acid amide hydrolase (FAAH) and monoacylglycerol lipase (MAGL), respectively [23].

**Figure 1:**
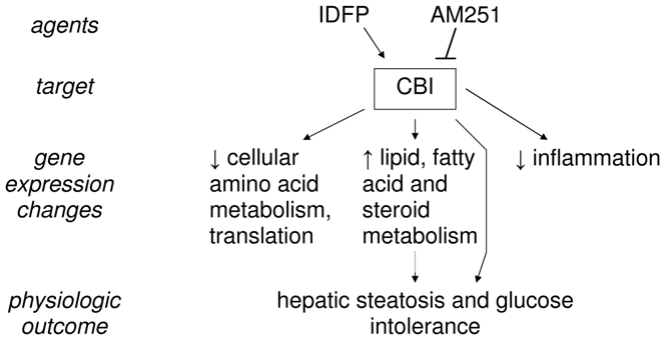
Effects of IDFP and AM251 on gene expression and physiological outcomes.

Since exogenously administered 2-AG and AEA are rapidly degraded [24], we have chosen to induce increases in their levels by inhibiting the enzymes responsible for their degradation. The organophosphorus (OP) compound isopropyl dodecylfluorophosphonate (IDFP) inhibits both MAGL and FAAH and raises 2-AG and anandamide levels *in vivo* [25], [26]. We have previously shown that IDFP increases circulating triglyceride (TG) concentrations in a CB1-dependent manner through the decreased clearance of TG-rich lipoproteins [26]. CB1 antagonists and knockout mice can be used to assess the contribution of CB1 signaling to IDFP effects. We describe here the CB1-dependent effects of IDFP on hepatic lipid content and insulin sensitivity. In addition, to gain mechanistic insight into CB1 regulation of lipid and glucose metabolism, we assayed global hepatic gene expression by microarray analysis of IDFP and AM251/IDFP-treated mice (Fig. 1).

## Materials and Methods

### Chemicals

AM251, the 4-iodophenyl analog of rimonabant that has a 4-chlorophenyl substituent, was obtained from Tocris Cookson Inc. IDFP was synthesized as previously described [27].

### Animals

This study was carried out in accordance with the recommendations in the Guide for the Care and Use of Laboratory Animals of the National Institutes of Health. The protocol was approved by the University of California-Berkeley Animal Care and Use Committee (Protocol R071-0310R). Injection of test compounds was performed under isoflurane anesthesia, and all efforts were made to minimize suffering. Swiss Webster mice were from Harlan Laboratories. CB1 +/+ and −/− breeding pairs were obtained from Andreas Zimmer and Carl Lupica [25], [28]. All experiments used Swiss Webster mice unless specifically stated otherwise, i.e. CB1 +/+ or −/−. All mice were 6–8 weeks of age, male, and weighed 18–23 g. Mice were fasted for 4 h, treated i.p. at 1 µl/g with dimethyl sulfoxide (DMSO) or test compounds dissolved in DMSO, and sacrificed 4 h after treatment with livers flash frozen. IDFP-treated mice in this study displayed cannabinoid-mediated behavioral effects similar to those previously reported [25].

### Biochemical Analyses

Flash frozen liver samples were homogenized in radioimmunoprecipitation assay buffer and TG and cholesterol were analyzed by enzymatic end-point measurements utilizing enzyme reagent kits (Catalog # F6428 & T2449, Sigma). Protein concentration of the hepatic homogenate was determined by the bicinchoninic acid method.

### Microarray Analysis

RNA was isolated from homogenized liver using the RNAeasy kit (Qiagen) and cRNA was synthesized from isolated RNA using the Illumina TotalPrep RNA amplification kit (Applied Biosystems). cRNAs from 19 individual mice were hybridized to mouseref-8 v2.0 beadchips and read on the iscan instrument. Group data represent the average of 5–8 individual mice randomized to three chips. Data were processed using BeadStudio software 3.2 (Illumina) with quantile normalization, background subtraction, and multiple testing correction (Benjamini and Hochberg false discovery rate). To quantify the effects of AM251 pre-administration relative to IDFP induced alterations a reversal metric was calculated as follows, with DMSO fold as 1.00:
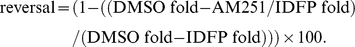
For example, lipocalin 2 (lcn2) has fold changes of 1.00 (DMSO), 0.20 (IDFP), and 2.68 (AM251/IDFP), thus percent reversal = (1–((1.00–2.68)/(1.00−.20)))×100 = 310%.

The web-based data analysis tools Panther (http://www.pantherdb.org) and FUNCASSOCIATE 2.0 ((http://llama.med.harvard.edu/funcassociate) were used with default settings. All microarray data is MAIME compliant and has been deposited in the GEO database (accession # GSE22949).

### PCR Confirmation

Relative quantitative PCR was performed on the ABI7900 system using SYBR green master mix in triplicate (Applied Biosystems). All genes were normalized to an endogenous control gene, gusb. The primers used are given in Supplemental Table S1.

### Glucose Tolerance and Insulin Sensitivity

Glucose (2 g/kg) was administered i.p. 2 h following treatment with IDFP or DMSO. Blood glucose was determined by the Onetouch ultra monitor system (Lifescan, Inc.) 0, 15, 30, 60, 90, 120 and 180 min following glucose administration. Plasma insulin was determined by ELISA (Millipore) 0, 10, 15, and 30 min following glucose administration. To test insulin sensitivity, insulin (0.5 U/kg) was administered i.p. 2 h following treatment with IDFP or DMSO and glucose was determined as above at 0, 15, 30, and 60 min following treatment.

### Statistical analyses

Results are presented as mean ± standard error. One- and two-way repeated measures analysis of variance was used to test significance of treatment effects. Post-hoc analysis (Tukey's) examined significance (p<0.05) of individual treatment effects. All analyses were performed using JMP version 7.0. Additional statistical information is given in Supplemental Table S2.

## Results

### 1.1 IDFP produces CB1-dependent hepatic steatosis

**Figure 2:**
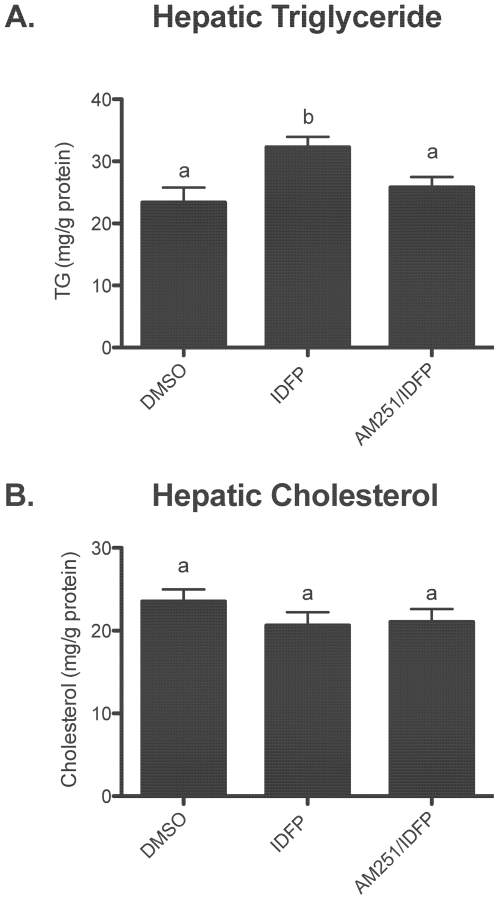
CB1-dependent effects of IDFP on hepatic TG (A) and cholesterol (B) levels. Mice were treated with DMSO or IDFP (10 mg/kg, ip, 4 h) alone or 15 min following AM251 (10 mg/kg, ip). n = 20–21. Groups not sharing a common superscript letter are significantly different (p<0.05).

To test the effects of elevated ECs on hepatic lipid levels, mice were treated with IDFP or vehicle control (DMSO). To determine the CB1-dependence of these effects, a subset of the IDFP group was pretreated with the CB1 antagonist, AM251. IDFP treatment significantly increased hepatic TG levels (Fig. 2A), while AM251 pre-administration partially reversed this effect (Fig. 2A). IDFP had no significant effect on hepatic cholesterol content (Fig. 2B). The partially CB1-dependent increase in hepatic TG but not cholesterol levels was confirmed in CB1 −/− mice and wild-type littermates treated with IDFP (Supplemental Fig. S1). These effects of cannabinoid signaling on hepatic triglyceride levels are consistent with previous studies showing cannabinoid agonists increase *de novo* lipogenesis and lipid accumulation in the liver [29], [30].

### 1.2 IDFP impairs glucose tolerance by both CB1-dependent and -independent effects

**Figure 3:**
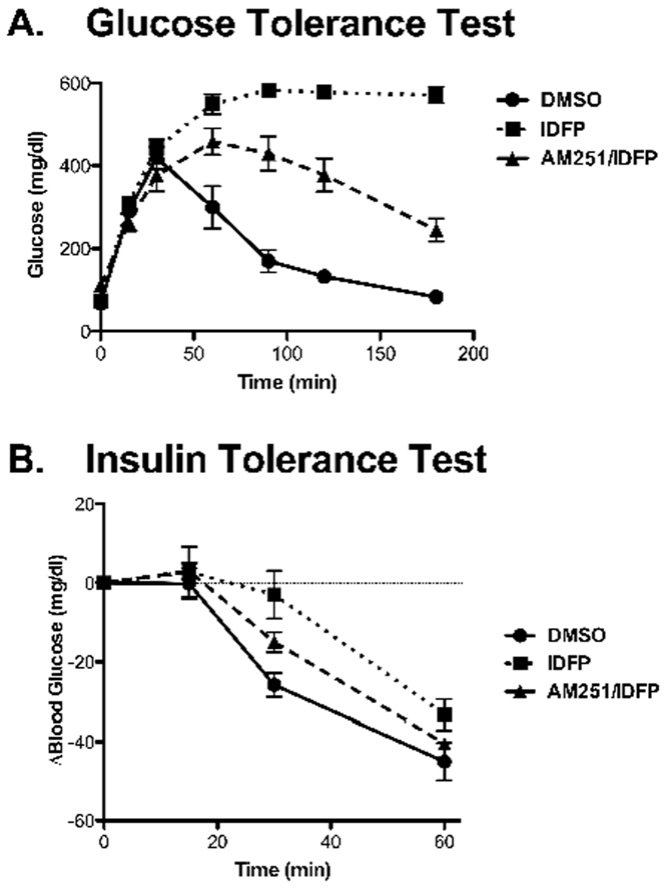
IDFP causes CB1-dependent and -independent glucose intolerance (A) and insulin resistance (B). Mice were treated with DMSO or IDFP (10 mg/kg, i.p., 4 h) alone or 15 min following AM251 (10 mg/kg, i.p.). Two h following DMSO or IDFP treatment, mice were administered glucose (2 g/kg) or insulin (0.5 U/kg) and plasma glucose was determined at the time points indicated. n = 4–5. Results were analyzed by repeated measures analysis of variance. The three groups responded in a significantly different fashion to glucose (F = 34.50, p = <0.0001), but not insulin (F = 1.96, p = .137).

To test the effect of MAGL/FAAH inhibition on glucose homeostasis, mice were treated with DMSO or IDFP with or without pre-administration of AM251 and then subjected to a glucose tolerance test. IDFP produced profound glucose intolerance (Fig. 3A). As the glucometer used in these studies had a maximum reading of 600 mg/dl, the glycemia of IDFP-treated animals may be underestimated, although 4 h after glucose administration the glucose levels of all animals were in the quantifiable range (data not shown). The IDFP effect was mitigated, but not completely reversed, by pre-administration of AM251 (Fig. 3A). Likewise, IDFP induced severe glucose intolerance in wild-type C57BL6 mice, but CB1 −/− littermates were less susceptible to IDFP induced glucose intolerance (Supplemental Fig. S2). Unexpectedly, the CB1 −/− mice were more glucose intolerant than their wild-type littermates. To test if the glucose intolerance resulted from insulin resistance or altered insulin secretion, insulin levels were determined 10,15, and 30 min after glucose administration in DMSO and IDFP-treated Swiss Webster mice. IDFP-treated mice had significantly elevated levels of insulin at all time points (10 min: 0.68±0.07 vs. 1.19±0.09 ng/ml, 15 min: 0.44±0.09 vs 1.29±0.26 ng/ml; 30 min: 0.42±0.03 vs 0.73±.08 ng/ml, all p<0.05, n = 5). As the elevated insulin levels suggest insulin resistance, an insulin tolerance test was performed to determine the effects of IDFP on insulin resistance. Although the effects did not reach statistical significance, IDFP decreased, while AM251 pretreatment partially restored, the glucose-lowering efficacy of insulin (Fig. 3B).

### 1.3 IDFP Alters Hepatic Gene Expression

#### 1.3.1 Cluster Analysis

**Figure 4:**
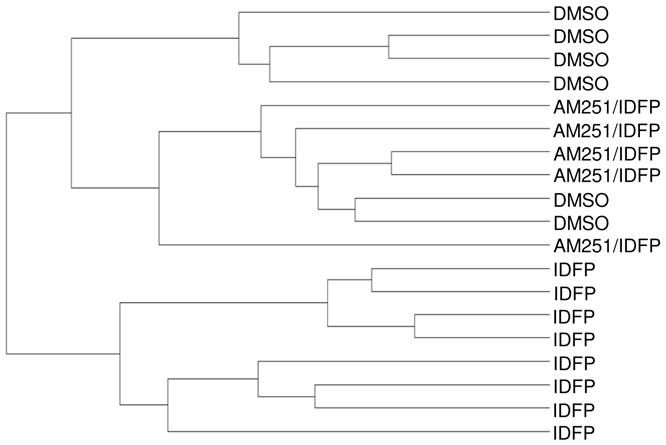
Hierarchical clustering of the transcriptome of individual mice. Dendrogram representation of cluster analysis from BeadStudio software 3.2 (Illumina).

**Figure 5:**
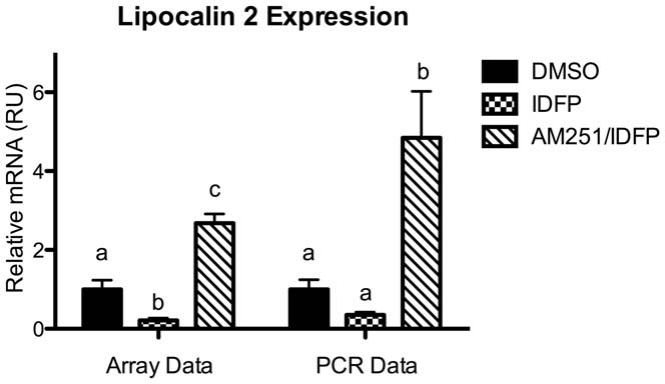
CB1-dependent effects of IDFP on hepatic expression of lipocalin 2. RNA was isolated from the mice used for the experiment in Fig. 2. Array n = 5–8. PCR n = 17–18. Groups within the array and PCR assays not sharing a common superscript letter are significantly different (p<0.05).

To identify the pathways activated by IDFP administration, specifically those dependent on CB1 activation, microarray analysis was performed on hepatic mRNA from DMSO, IDFP and AM251/IDFP-treated animals. Of the 18,097 genes present on the array, 8,857 were detected in at least one of the groups at p<0.05. IDFP-treated animals segregated well from DMSO treated animals in cluster analysis, while results for the AM251/IDFP-treated animals were intermingled with those for the DMSO treated animals (Fig. 4). After correction for multiple testing, the expression of 533 genes was significantly altered by IDFP administration (Supplemental Table S3). Of these, 230 were increased by IDFP, while 303 were decreased. These 533 genes had a skewed distribution of p-values for the IDFP vs. AM251/IDFP comparison, making them inappropriate for the generation of q-values and false discovery rate analysis. To quantitate the ability of AM251 pre-administration to reverse IDFP-induced changes in gene expression, the percent reversal of the 533 significantly altered genes was determined (see methods for calculations). Although there was a large range (−128 to 310%), the average percent reversal (58±2%) was high, indicating that AM251 reversed a significant portion of the IDFP effects on gene expression. Lipocalin 2 (lcn2) had the highest percent reversal (310%), with IDFP decreasing and AM251 pre-administration increasing expression levels, effects confirmed by PCR (Fig. 5).

#### 1.3.2 Identification of Functional Categories

Two complementary approaches were used to identify functional categories within the data set. First, the Panther classification system was used to determine biological processes regulated within the dataset. This analysis accounts for the fold change of every gene detected, but is limited to differential analysis of two groups at a time. Lipid, fatty acid, and steroid metabolism was the only category identified when IDFP was compared with both DMSO and AM251/IDFP (Supplemental Table S4). To identify a subset of genes with significantly altered CB1-dependent expression, upregulated (n = 112) and downregulated (n = 168) genes with >50% reversal and a nominal p value <0.05 between the IDFP and IDFP/AM251 groups were queried in Funcassociate 2.0. Although the 50% reversal cutoff for inclusion of a gene as CB1-dependent was arbitrary, stricter thresholds (80% cutoff) yielded similar results in the pathway analysis. While the upregulated genes were not significantly enriched for any gene ontology attributes, the downregulated genes were enriched in genes with roles in translation, cellular amino acid metabolic processes, and the acute phase response (Supplemental Table S5). The altered CB1-dependent genes represented within the enriched pathways and biological processes are shown in Table 1.

**Table 1:** Altered CB1-dependent Genes Represented within the Enriched Pathways.

Pathway	Fold Change vs. DMSO^a^ (1.00)		Reversal by AM251^b^ (%)
	IDFP	AM251/IDFP	
**Acute Phase Response**			
orm1	0.70	1.43	246
orm2	0.41	1.79	233
saa2	0.25	1.30	139
saa4	0.51	1.11	123
stat3	0.52	1.01	102
**Amino Acid Metabolism & Translation**			
Rars	0.74	1.12	147
Lars	0.63	1.11	129
Aars	0.65	1.00	100
Iars	0.65	0.97	93
Farsb	0.74	0.96	84
rars2	0.62	0.87	66
Gars	0.65	0.85	58
ncoa5	0.57	0.81	55
eef1e1	0.57	1.14	133
eif2b4	0.73	0.95	82
psat1	0.29	0.85	79
Pelo	0.64	0.91	75
Asns	0.15	0.68	62
eif2ak2	0.51	0.80	59
**Lipid, Fatty Acid, and Steroid Metabolism**			
sgms2	2.00	0.60	140
pgc1β	6.78	2.16	80
pla2g6	1.68	1.30	55
mtmr3	2.08	1.42	61
lpin2	3.22	1.70	69
insig1	1.90	1.08	91
chkb	1.81	1.26	67
apol9b	1.66	1.02	97
acsl1	1.79	1.05	93
ldlr	4.50	1.30	92

a Average fold change for 5 (DMSO), 8 (IDFP), and 7 (AM251/IDFP) individual mice.

b See methods for calculation of reversal by Am251.

#### 1.3.3 PCR Confirmation

**Figure 6:**
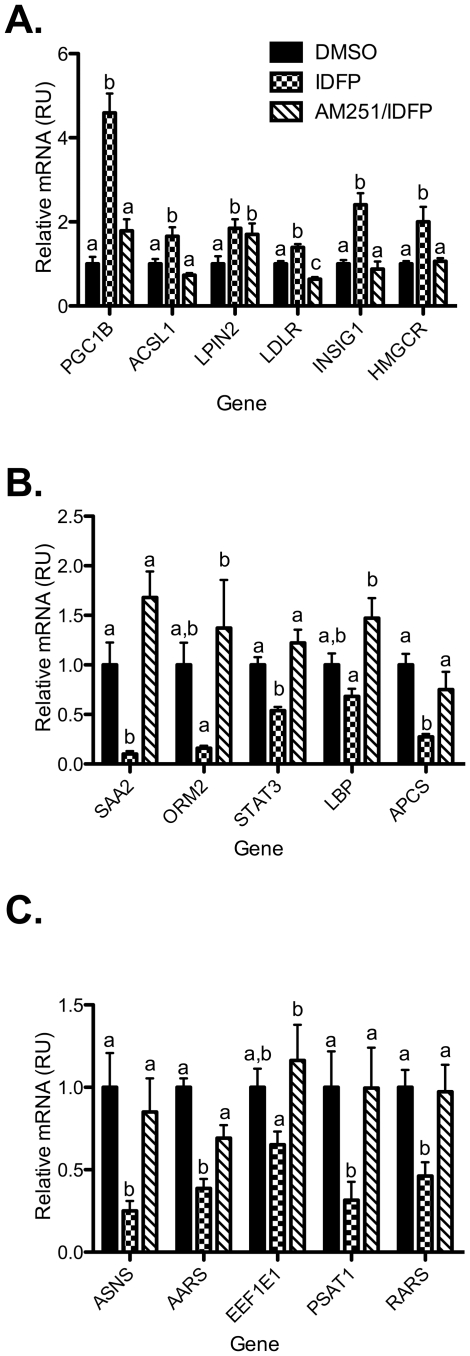
CB1-dependent effects of IDFP on hepatic expression of genes involved in lipid metabolism (A), inflammation (B), and amino acid metabolism (C). RNA was isolated from the mice used for the experiment in Fig. 2. n = 16–18. Groups not sharing a common superscript letter are significantly different (p<0.05).

To confirm the effects of IDFP on hepatic gene expression, quantitative PCR was performed on a larger set of samples representing three independent experiments, including the one used for the array data. The results confirmed increased expression of several key components of the lipid and cholesterol metabolism gene set including ldlr, insig1, pgc1β, lpin2, and acsl1 (Fig. 6A). With the exception of lpin2, the CB1-dependence of these effects was shown by their reversal by AM251. Given the effects of IDFP on the SREBP2 target genes, insig1 and ldlr, we also chose to probe the effect of IDFP on expression of hmgcr which encodes the rate-limiting enzyme in cholesterol biosynthesis, HMGCoA reductase, and which was not represented on the array. IDFP increased expression of hmgcr, an effect that was completely prevented by AM251 pre-administration (Fig. 6A).

The PCR results confirmed CB1-dependent inhibitory effects of IDFP on several acute phase (saa2, orm2, stat3) and amino acid response genes (asns, aars, eef1e1, psat1, rars) (Fig. 6B,C). As stat3 is an essential transcription factor involved in the acute phase response, we tested the effect of IDFP on the canonical stat3 downstream targets apcs and lbp [31]. IDFP decreased expression of both apcs and lbp in a CB1-dependent manner. There was a strong correlation between the array and PCR data for all genes tested (r = 0.7–1.0). The effects of IDFP on expression of genes involved in lipid metabolism and stat3 signaling were determined in CB1 −/− and wildtype littermates. There was a significant genotype-treatment interaction for several of the lipid metabolism genes tested, (ldlr, insig1, pgc1β, and acsl1), confirming a CB1-dependent effect of IDFP (Supplemental Table S6). Stat3 and its targets, lbp and apcs, displayed the expected trend, but the results did not reach statistical significance (Supplemental Table S6).

## Discussion

The data presented here establish that IDFP induces hepatic steatosis and insulin resistance partially through CB1 signaling. IDFP significantly increased hepatic TG levels 4 h after treatment. This effect was largely mediated by the CB1 receptor, as it was mitigated by AM251 pre-administration and deficient in CB1 −/− mice. As neither the effects of AM251 nor the genetic absence of CB1 in the animals used in the current study are confined to the periphery, the site of CB1 action responsible for the observed effects cannot be addressed. However, all animals were fasted prior to and during the experimental period eliminating differential energy intake as a confounding variable. Hepatic TG content reflects the balance of secretion, uptake, synthesis, and oxidation. We have previously shown that IDFP decreases clearance of circulating TGs with no effect on hepatic TG secretion [26]. The IDFP-induced increase in hepatic TG content combined with a lack of effect on TG secretion suggests that CB1 stimulation may favor TG storage over secretion. Correspondingly, treatment with a non-brain-penetrant CB1 antagonist has been reported to reduce hepatic steatosis and increase secretion of TG-rich lipoproteins [22]. Since CB1 antagonists increase adipose TG lipolysis, CB1 stimulated hepatic TG accumulation likely does not result from increased delivery of FA from adipose tissue [16], [32]. Hepatic CB1 stimulates sprebp1c and inhibits AMP kinase making both decreased oxidation and increased synthesis of fatty acids likely contributors to the IDFP induced increase in hepatic TG content [20], [21]. Indeed, we found that IDFP increases expression of the lipogenic genes, fas and srebp1c, in a CB1-dependent manner [26]. The CB1 independent effects of IDFP on hepatic TGs may result in part from lipolytic defects resulting from MAGL blockade [33], other monoacylglycerol or N-acylethanolamine or N-acyltaurine substrates of MAGL and FAAH [34], [35], respectively, or off-target effects of IDFP [25]. Further studies with specific MAG lipase inhibitors and knockout mice will be necessary to clarify the role of MAG lipase in the lipolytic cascade.

We have shown that IDFP causes profound glucose intolerance. Our results are consistent with previous reports that synthetic CB1 agonists caused glucose intolerance and insulin resistance in wild-type but not in hepatocyte-specific CB1 knockout mice [21]. Conversely, genetic or pharmacological inactivation of CB1 prevents diet-induced alterations in glucose and insulin tolerance [21]. In humans, Δ^9^-tetrahydrocannabinol (THC) has been shown to cause glucose intolerance [36]. Although a CB1-dependent effect of IDFP on glucose intolerance is indicated by our finding that this was substantially prevented by AM251, there were also CB1-independent effects of IDFP. Similarly, CB1 knockout mice were less susceptible to IDFP induced glucose intolerance, but still had a significant response.

Glucose tolerance reflects the ability of insulin to drive glucose uptake and shut down glucose production. In the present study, the elevated insulin levels in IDFP-treated animals following glucose administration indicate that the glucose intolerance likely results from severe insulin resistance. Glucose response during an insulin tolerance test largely represents the ability of insulin to inhibit glucose production in the liver. Our results suggest that although some reduction of hepatic insulin sensitivity may have been induced by IDFP, this is likely not sufficient to have produced the profound glucose intolerance that was observed. There may also be effects of ECs on peripheral glucose uptake. CB1 antagonist administration increases muscle glucose uptake muscle in ob/ob mice [37]. EC levels have also been shown to be higher in the muscle of diet-induced obese mice [5]. Physical inactivity in IDFP-treated mice may also contribute indirectly to reduced glucose utilization [25]. Although we did not observe changes in resting glucose levels, this may be due to the acute nature of the IDFP treatment regimen used in this study. Whether chronic EC elevation leads to fasting hyperglycemia warrants further investigation.

The CB1-independent effects of IDFP on glucose tolerance are shared by other OP compounds [38]. Instances of OP poisoning have been characterized by marked hyperglycemia, glycosuria, and diabetic ketosis [39], [40], [41]. Acetylcholinesterase (AChE) inhibition has been proposed to underlie OP-induced hyperglycemia [42]. However, since IDFP does not inhibit AChE *in vivo*, IDFP, and perhaps other OPs, produce glucose intolerance via CB1 and AChE independent pathways.

Our findings suggest that increased CB1 signaling can be considered a cause rather than a consequence of hepatic steatosis and insulin resistance. As only a few genes have been identified as CB1 responsive in the liver, we aimed to identify novel CB1 targets stimulated by endocannabinoids. Although the microarrays detected ∼9000 genes in at least one of the sample groups, certain genes were not represented on the chip, such as hmgcr. PCR was used to validate several of the array findings in multiple independent experiments.

Hepatic CB1 has been shown to increase expression of the lipogenic genes fas, acc, and srebp1c [30]. Here we identify pgc1β as a novel downstream target of CB1. This is of particular interest as PGC1β is a co-activator for SREBP1c and is necessary for the development of diet-induced hyperlipidemia [43]. Likewise expression of acsl1 was stimulated by CB1 activation. Acyl-CoA synthetases activate fatty acids into acyl-CoAs, providing substrates for downstream fatty acid metabolic processing, such as esterification and beta-oxidation. Acsl1 is highly expressed in the liver and hepatocyte specific loss of acsl1 has been shown to decrease both fatty acid oxidation and incorporation into TG [44]. Acsl1 overexpression enhances incorporation of fatty acids in diacylglycerol, but does not cause TG accumulation [45]. The effect of raising pgc1β and acsl1 expression in the setting of CB1 signaling requires further study.

Several of the key regulators of cholesterol homeostasis were increased by IDFP in a CB1-dependent manner. This is of particular interest as CB1 signaling regulates plasma lipid and lipoprotein metabolism. In clinical trials, rimonabant had greater effects on TG and HDL cholesterol than would be expected based on weight loss alone [12]. Hepatocyte specific CB1 −/− mice are immune to diet induced alterations in plasma lipoproteins and we have previously shown that IDFP causes hypertriglyceridemia secondary to decreased clearance of TG rich lipoproteins [21], [26]. The coordinated regulation of several SREBP2 target genes by IDFP in a CB1-dependent manner may result from direct regulation of this pathway by the CB1 signaling cascade. Alternatively, the IDFP induced decreased uptake of plasma lipoproteins may transiently decrease hepatic cholesterol content, triggering SREBP2 activity. Although we did not detect alterations in hepatic cholesterol content, the small magnitude and transience of changes required to stimulate SREBP2 processing may not be detectable by the present methodology.

There is a complex relationship between CB1 signaling and inflammation. Both activation and inhibition of CB1 have been shown to have anti-inflammatory outcomes in different contexts [46], [47], [48], [49]. In our study, IDFP decreased expression of genes encoding acute phase proteins in a CB1-dependent manner. Interestingly, stat3 and several of its targets (apcs, lbp) were downregulated in livers of IDFP-treated mice. In neurons CB1 activation stimulates stat3 [50]. Consistent with a reversal of this system in the liver, rimonabant administration was shown to potentiate stat3 activation in response to lipopolysaccharide stimulation [46]. While our results establish that CB1 stimulation limits hepatic inflammation at the mRNA level under basal conditions, the role of CB1 on systemic inflammation in response to various stimuli remains to be determined.

Inflammatory signaling pathways, including stat3, influence metabolic regulation. STAT3 signaling mediates the hypophagic and hypoglycemic effects of leptin. Additionally, STAT3 downregulates srebp1c which was increased by IDFP in a CB1-dependent manner [51], [52]. Hepatocyte specific loss of stat3 produces insulin resistance and increases susceptibility to ethanol induced hepatic steatosis, lipogenic gene expression, and hypertriglyceridemia [51], [53]. Findings with a non-brain-penetrant CB1 antagonist suggest that diet-induced leptin resistance may result from overactive peripheral CB1 signaling [22]. The current study raises the possibility that CB1 mediated inhibition of hepatic STAT3 signaling may influence leptin sensitivity.

LCN2 is an acute phase protein known to regulate energy and glucose metabolism [54], [55]. Although LCN2 is a member of the lipocalin sub-family of secreted proteins that bind hydrophobic molecules, its specific ligands remain largely unknown [56]. Lcn2 knockout animals are cold intolerant and more susceptible to diet-induced increases in plasma lipids and adipose mass [55], [57]. Reports on insulin sensitivity and hepatic lipid content in lcn2 deficient mice are discordant with one group reporting protective and another potentiating effects [55], [57]. In our study, IDFP inhibited expression of hepatic lcn2. Interestingly, AM251 pre-administration not only prevented the effects of IDFP, but also increased lcn2 expression compared to controls. This suggests that tonic endogenous CB1 stimulation may limit lcn2 expression. EC binding to lipocalins has been suggested to facilitate their release from the plasma membrane and subsequent delivery to targets [58]. If this is the case, the robust regulation of lcn2 by CB1 observed here may represent a feedback cycle. Alternatively, we cannot exclude the possibility that this effect could be independent of ECs and may reflect the inverse agonist properties of AM251. Future studies will be necessary to clarify the relationship between lcn2 and CB1 signaling.

Although CB1 signaling has been linked to ER stress and mammalian target of rapamycin (mTOR) activity, the relationship between ECs and amino acid metabolism remains unclear. mTOR is a serine/threonine protein kinase central to regulation of amino acid metabolism and translation. THC, in a CB1-dependent fashion, stimulates mTOR in the hippocampus, but inhibits mTOR by stimulating ER stress in cancer cells [59], [60]. Recently, administration of a non-brain-penetrant CB1 antagonist has been found to reverse ER stress caused by a high fat diet [22]. In our study of hepatic tissue gene expression, IDFP caused a CB1-dependent decrease in the gene cassette involved in translation and amino acid metabolism.

As IDFP inhibits both FAAH and MAGL, the CB1-dependent effects cannot be specifically ascribed to increases in either AEA or 2-AG levels. Animals deficient in FAAH, with elevated AEA but not 2-AG, have increased body weight, tissue TG content, fasting blood glucose and insulin compared to controls when fed a high fat diet, without differences in food intake [61]. The use of specific chemical inhibitors or genetic manipulation of MAGL and/or FAAH will be necessary to establish the relative contribution of each enzyme to the effects shown here. The simultaneous elevation of AEA and 2-AG has been shown to have synergistic effects that are not recapitulated by raising levels of either EC alone [34]. Moreover, synthetic CB1 agonists may bind the receptor differently and produce differential downstream responses than the ECs. Nevertheless, study of the effects of synthetic CB1 agonists on expression of the genes found here to be responsive to IDFP, and the reversal of these effects by AM251, would be informative.

IDFP is perhaps the most potent dual FAAH/MAGL inhibitor reported to date and although it does not reduce AChE activity there could be other off target effects that may potentiate the IDFP-induced metabolic effects observed in this study [25], [27] (Supplemental Table S7). The carbamate FAAH/MAGL inhibitor JZL195 displays better specificity in the brain, but its selectivity in other tissues has not yet been established [54]. It is likely to have additional off-targets similar to those of the structurally-related MAGL-selective inhibitor JZL184 [62]. Our previous studies have shown that AM251 completely reverses all cannabinoid-mediated behavioral effects observed with IDFP or direct CB1 agonists such as WIN55212-2 [25]. We have therefore restricted our interpretations to the IDFP-effects that are reversible with cannabinoid receptor antagonism.

While the current study addresses the acute metabolic effects of dual MAGL/FAAH inhibition, the chronic metabolic effects of such inhibition requires further investigation. Genetic ablation of FAAH promotes increases in body weight, adipose tissue amount, plasma free fatty acids and triglyceride content in plasma, liver, skeletal muscle, and adipose tissue [61]. On the other hand, as MAGL deficient mice have reduced sensitivity to CB1 agonists and attenuated diet-induced insulin resistance, chronic elevation of 2-AG may lead to functional antagonism of cannabinoid receptors potentially negating the effects of elevations in 2-AG [33], [63].

In summary, our results support a causative role of CB1 signaling in the development of hepatic steatosis and insulin resistance. Furthermore, we have identified novel genes responsive to IDFP in a CB1-dependent manner that may guide future research on CB1-mediated modulation of pathways impacting lipid and glucose metabolism.

## Supporting Information

Figure S1 **CB1-dependent effects of IDFP on hepatic TG (A) and cholesterol (B) levels.** Wild-type and CB1 −/− mice were treated with DMSO or IDFP (10 mg/kg, ip, 4 h). n = 5–6. Significance is given as *p<0.05.
(TIFF)

Figure S2 **IDFP causes CB1-dependent and-independent glucose intolerance.** Wild-type (A) and CB1 −/− (B) mice were treated with DMSO or IDFP (10 mg/kg, ip, 4 h). Two h following DMSO or IDFP treatment, mice were administered glucose (2 g/kg) and plasma glucose determined at the time points indicated. n = 5.
(TIFF)

Table S1 **PCR Primers** Sequence of primers used to amplify transcripts in PCR.
(DOCX)

Table S2 **Additional Statistical Detail** Additional statistical details of one- and two-way ANOVAs used to determine significance.
(DOCX)

Table S3 **Genes Significantly Altered by IDFP** Genes determined to be significantly altered by IDFP after multiple testing correction.
(DOCX)

Table S4 **Panther Biological Process Analysis** Biological processes found to be significantly altered by Panther analysis.
(DOCX)

Table S5 **FuncAssociate 2.0 Analysis of Genes Decreased by IDFP in a CB1 Dependent Manner** Summary of FuncAssocaite analysis of genes (n = 168) downregulated by IDFP and reversed by Am251(>50%).
(DOCX)

Table S6 **Expression of Genes involved in Stat3 signaling and Lipid Metabolism in WT and CB1 −/− Mice** Wild-type and CB1 −/− mice were treated with DMSO or IDFP (10 mg/kg, ip, 4 h). Groups not sharing a common superscript letter are significantly different (p<0.05). n = 5.
(DOCX)

Table S7 **Known off target effects of IDFP** Enzymes known to be altered by IDFP.
(DOCX)
