# Supplementary figures and images for: Acute Overactive Endocannabinoid Signaling Induces Glucose Intolerance, Hepatic Steatosis, and Novel Cannabinoid Receptor 1 Responsive Genes
Source: PLoS One. 2011 Nov 4;6(11):e26415. doi: 10.1371/journal.pone.0026415 (PMC3208546; doi:10.1371/journal.pone.0026415)

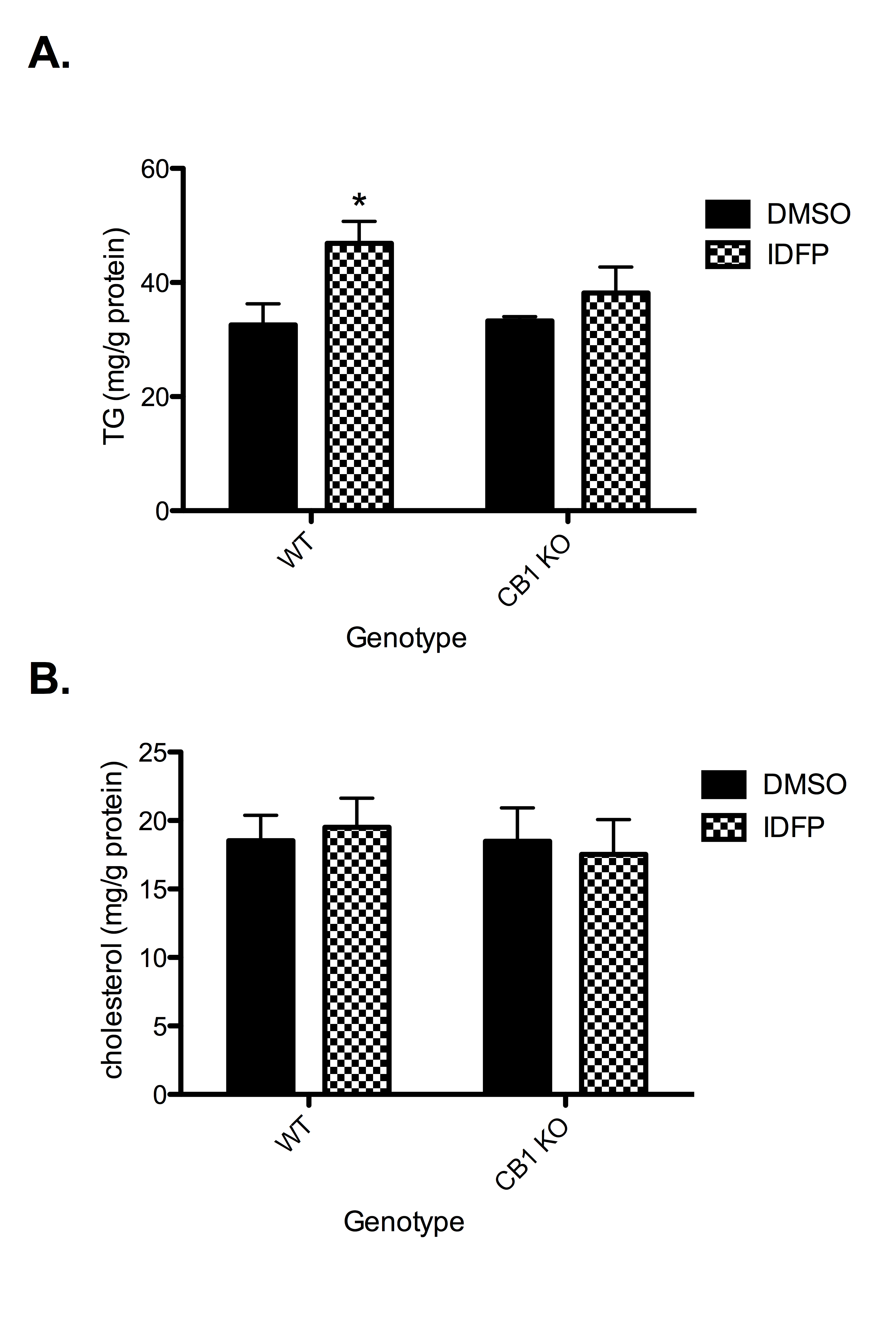

Supplement: Figure S1 — CB1-dependent effects of IDFP on hepatic TG (A) and cholesterol (B) levels. Wild-type and CB1 −/− mice were treated with DMSO or IDFP (10 mg/kg, ip, 4 h). n = 5–6. Significance is given as *p<0.05. (TIFF) [file pone.0026415.s001.tif]

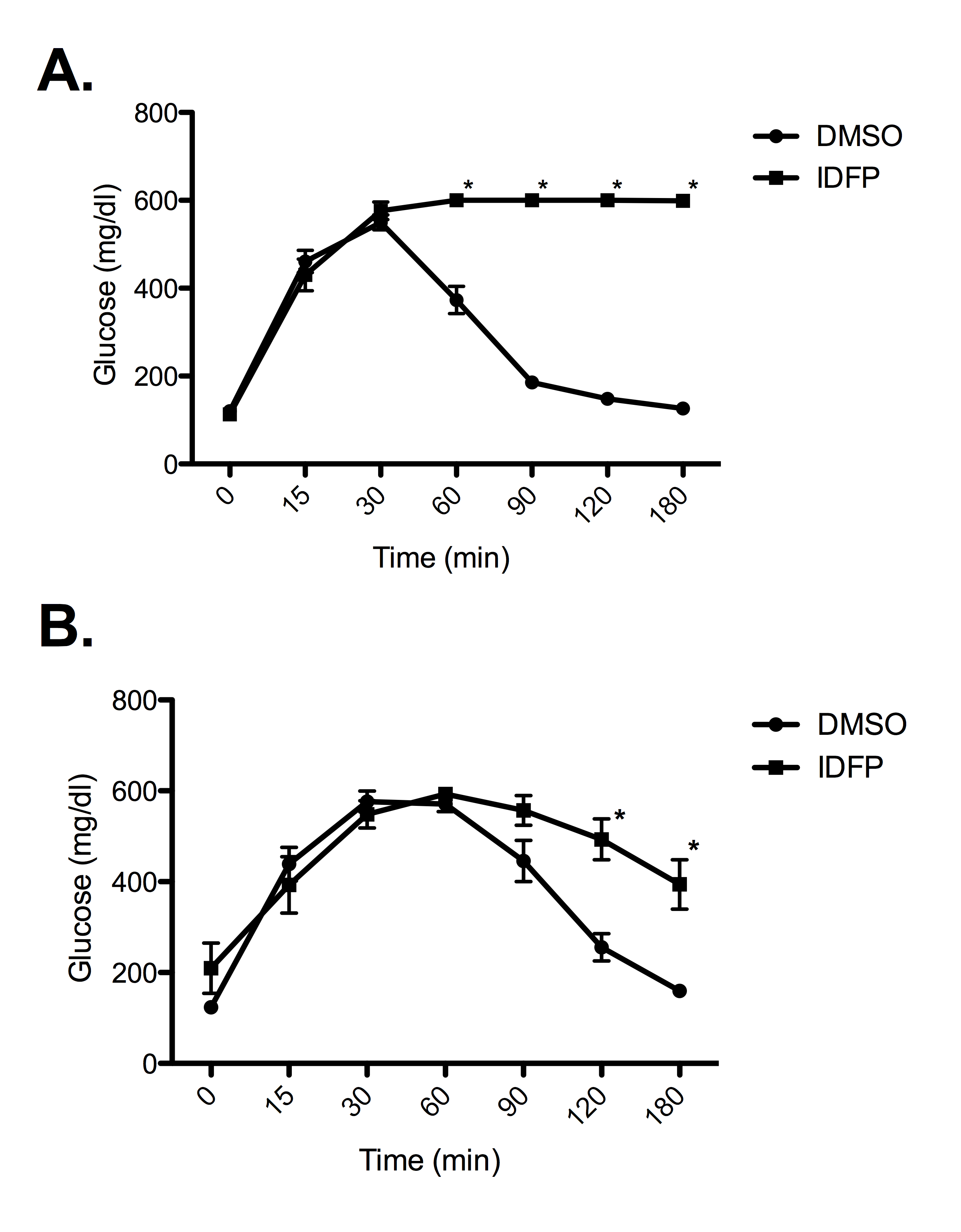

Supplement: Figure S2 — IDFP causes CB1-dependent and-independent glucose intolerance. Wild-type (A) and CB1 −/− (B) mice were treated with DMSO or IDFP (10 mg/kg, ip, 4 h). Two h following DMSO or IDFP treatment, mice were administered glucose (2 g/kg) and plasma glucose determined at the time points indicated. n = 5. (TIFF) [file pone.0026415.s002.tif]
