# Supplementary material for: Acute Overactive Endocannabinoid Signaling Induces Glucose Intolerance, Hepatic Steatosis, and Novel Cannabinoid Receptor 1 Responsive Genes
Source: PLoS One. 2011 Nov 4;6(11):e26415. doi: 10.1371/journal.pone.0026415 (PMC3208546; doi:10.1371/journal.pone.0026415)
Supplement: Table S1 — PCR Primers Sequence of primers used to amplify transcripts in PCR. (DOCX) [file pone.0026415.s003.docx]

**Supplemental Table 1: PCR Primers**

|  | Forward | Reverse |
| --- | --- | --- |
| gusb | CAT GAG AGT GGT GTT GAG GAT CA | CCC ATT CAC CCA CAC AAC TG |
| orm2 | TCA TGC TTG CCT TTG ACT TG | CAC GTG TGT GAC AGC CTT CT |
| psat1 | AGC TCA GCT CCA TCA AAT CC | CAA AGC TTC GTC TCC TTT GG |
| eef1e1 | AAA GGC AAT GGT TCA GCA GT | CGC CAG GGT GAT GTT ATG T |
| aars | TGG AGT GCA GAC AGA TTT GG | TCA CCC ATC TCC CAG AAG TT |
| rars | TTG CTG CTG CTC AGA TGA TT | CAT AAG GCG CAC AGT TTC AC |
| asns | GGG CAG AGA CAC CTA TGG AG | GAA GGA AGG GCTCCA CTT TT |
| acsl | CAG TTC ATC GGC CTC TTC TC | TCA GCT CCA AGG GTG TCA TA |
| pgc1b | GAG CTT TGA GGA GTC CCT GA | GGC TTG TAT GGA GGT GTG GT |
| ldlr | ACA GTG GCG TCA GTG ACA GT | CTC ATA CCA TGT GGC TGC TC |
| hmgcor | CTG GTG AGC TGT CCT TGA TG | GCG CTT CAG TTC AGT GTC AG |
| lbp | TCA CAC TAC CGG ACT TCA GC | GGA GCA GCT TCA GAG AGG AG |
| stat3 | CCC GTA CCT GAA GAC CAA GT | GCA CCT TCA CCG TTA TTT CC |
| apcs | TGG ACA AGC TAC TGC TTT GG | GAT GTG GGA TCA GCT TCA CA |
| saa | GGG GAA CTA TGA TGC TGC TC | TGG TGT CCT CAT GTC CTC TG |
| insig1 | CTG TAT TGC CGT GTT CGT TG | CTT CGG GAA CGA TCA AAT GT |
| lpin2 | CTA TGC TGC CTT TGG AAA CC | CGA TGA TTT GTT CCC TTT GG |
| lcn2 | CCA GTT CGC CAT GGT ATT TT | GGG TGA AAC GTT CCT TCA GT |
