# Supplementary material for: Acute Overactive Endocannabinoid Signaling Induces Glucose Intolerance, Hepatic Steatosis, and Novel Cannabinoid Receptor 1 Responsive Genes
Source: PLoS One. 2011 Nov 4;6(11):e26415. doi: 10.1371/journal.pone.0026415 (PMC3208546; doi:10.1371/journal.pone.0026415)
Supplement: Table S2 — Additional Statistical Detail Additional statistical details of one- and two-way ANOVAs used to determine significance. (DOCX) [file pone.0026415.s004.docx]

**Supplemental Table 2: Additional Statistical Detail**

|  | **Degrees of Freedom** | | **F Value** | **Treatment Effect** | **Post-Hoc Analysis (Tukey’s) P Values** | | |
| --- | --- | --- | --- | --- | --- | --- | --- |
|  | Between Group | Within Group |  | P Value | DMSO vs IDFP | DMSO vs AM251/IDFP | IDFP vs AM251/IDFP |
| **Figure 2** |  |  |  |  |  |  |  |
| A. Hepatic TG | 2 | 59 | 6.09 | 0.0039 | <0.01 | ns | <0.05 |
|  |  |  |  |  |  |  |  |
| **Figure 3** |  |  |  |  |  |  |  |
| A. GTT | 2 | 12 | 34.50 | 0.0001 | <0.01 | <0.01 | <0.01 |
|  |  |  |  |  |  |  |  |
| **Figure 5** |  |  |  |  |  |  |  |
| LCN2 Array | 2 | 16 | 51.30 | 0.0001 | <0.05 | <0.01 | <0.01 |
| LCN2 PCR | 2 | 48 | 11.18 | 0.0001 | ns | <0.01 | <0.01 |
|  |  |  |  |  |  |  |  |
| **Figure 6** |  |  |  |  |  |  |  |
| A. Lipid Metabolism | 2 | 48 |  |  |  |  |  |
| PGC1B |  |  | 33.44 | 0.0001 | <0.01 | ns | <0.01 |
| ACSL1 |  |  | 7.82 | 0.0011 | <0.05 | ns | <0.01 |
| LPIN2 |  |  | 4.27 | 0.0194 | <0.05 | ns | ns |
| LDLR |  |  | 14.67 | 0.0001 | <0.05 | <0.05 | <0.01 |
| INSIG1 |  |  | 19.13 | 0.0001 | <0.01 | ns | <0.01 |
| HMGCoAR |  |  | 6.80 | 0.0024 | <0.01 | ns | <0.01 |
| B. Inflammation | 2 | 48 |  |  |  |  |  |
| SAA2 |  |  | 6.24 | 0.0043 | <0.05 | ns | <0.01 |
| ORM2 |  |  | 4.67 | 0.0138 | ns | ns | <0.05 |
| STAT3 |  |  | 14.48 | 0.0001 | <0.01 | ns | <0.01 |
| LBP |  |  | 7.38 | 0.0013 | ns | ns | <0.01 |
| APCS |  |  | 9.22 | 0.0004 | <0.01 | ns | <0.05 |
| C. Amino Acid Metabolism | 2 | 48 |  |  |  |  |  |
| ASNA |  |  | 5.25 | 0.0086 | <0.05 | ns | <0.05 |
| AARS |  |  | 9.87 | 0.0003 | <0.01 | ns | <0.05 |
| EEF1E1 |  |  | 4.26 | 0.0204 | ns | ns | <0.05 |
| PSAT1 |  |  | 5.32 | 0.0081 | <0.05 | ns | <0.05 |
| RARS1 |  |  | 5.73 | 0.0059 | <0.05 | ns | <0.05 |
