# Supplementary material for: Acute Overactive Endocannabinoid Signaling Induces Glucose Intolerance, Hepatic Steatosis, and Novel Cannabinoid Receptor 1 Responsive Genes
Source: PLoS One. 2011 Nov 4;6(11):e26415. doi: 10.1371/journal.pone.0026415 (PMC3208546; doi:10.1371/journal.pone.0026415)
Supplement: Table S3 — Genes Significantly Altered by IDFP Genes determined to be significantly altered by IDFP after multiple testing correction. (DOCX) [file pone.0026415.s005.docx]

**Supplemental Table 3: Genes Significantly Altered by IDFP**

| **SYMBOL** | **ENTREZ**  **GENE ID** | **Fold Change vs. DMSO (1.00)** | | **Nominal P-Values** | | | **Reversal**  **by Am251**  **(%)** |
| --- | --- | --- | --- | --- | --- | --- | --- |
|  |  | **IDFP** | **AM251/IDFP** | **DMSO vs. IDFP** | **IDFP**  **vs.**  **AM251**  **/IDFP** | **DMSO**  **vs. AM251**  **/IDFP** |  |
| 0610040J01Rik | 76261 | 1.40 | 1.05 | 0.0033 | 0.0116 | 0.4560 | 87 |
| 1110002N22Rik | 68550 | 0.61 | 0.82 | 0.0002 | 0.0128 | 0.0328 | 54 |
| 1190005F20Rik | 98685 | 0.45 | 0.59 | 0.0000 | 0.0816 | 0.0031 | 26 |
| 1200014J11Rik | 66874 | 0.72 | 0.74 | 0.0007 | 0.7028 | 0.0052 | 6 |
| 1300001I01Rik | 74148 | 1.42 | 1.24 | 0.0001 | 0.1297 | 0.0646 | 43 |
| 1300007L22Rik | n/a | 0.70 | 0.90 | 0.0074 | 0.1727 | 0.5277 | 66 |
| 1700030K09Rik | 72254 | 0.41 | 0.46 | 0.0014 | 0.2304 | 0.0171 | 9 |
| 1700123O20Rik | 58248 | 0.64 | 0.96 | 0.0000 | 0.0001 | 0.5039 | 88 |
| 1810008A18Rik | 108707 | 0.60 | 0.69 | 0.0002 | 0.1440 | 0.0108 | 22 |
| 1810015C04Rik | 66270 | 1.84 | 2.41 | 0.0009 | 0.0290 | 0.0001 | -68 |
| 2010011I20Rik | 67017 | 1.57 | 1.19 | 0.0021 | 0.0139 | 0.1920 | 67 |
| 2010305A19Rik | 69893 | 0.33 | 0.58 | 0.0000 | 0.0013 | 0.0034 | 37 |
| 2210412D01Rik | 70178 | 1.48 | 1.09 | 0.0067 | 0.0283 | 0.3647 | 81 |
| 2310016C08Rik | 69573 | 2.82 | 1.48 | 0.0011 | 0.0253 | 0.0984 | 73 |
| 2310044G17Rik | 217732 | 0.72 | 0.96 | 0.0056 | 0.0063 | 0.6385 | 85 |
| 2310065K24Rik | 102122 | 0.60 | 0.82 | 0.0010 | 0.0014 | 0.1847 | 55 |
| 2410002O22Rik | 66975 | 0.59 | 0.91 | 0.0000 | 0.0002 | 0.1297 | 77 |
| 2700097O09Rik | 72658 | 0.58 | 1.03 | 0.0000 | 0.0000 | 0.6330 | 107 |
| 2810021B07Rik | 66308 | 0.57 | 0.64 | 0.0002 | 0.3895 | 0.0040 | 16 |
| 2810403A07Rik | 74200 | 0.59 | 0.76 | 0.0003 | 0.1108 | 0.0470 | 40 |
| 3110001A13Rik | 66540 | 1.78 | 1.30 | 0.0012 | 0.0294 | 0.0981 | 62 |
| 3110037I16Rik | 73172 | 0.55 | 0.62 | 0.0018 | 0.5287 | 0.0145 | 15 |
| 3300001P08Rik | 67684 | 0.51 | 0.79 | 0.0000 | 0.0055 | 0.0673 | 56 |
| 4833426J09Rik | 382051 | 1.37 | 1.37 | 0.0034 | 0.9701 | 0.0641 | -2 |
| 4932442K08Rik | 67544 | 0.31 | 0.61 | 0.0003 | 0.0478 | 0.0662 | 43 |
| 4933411K20Rik | 66756 | 0.65 | 0.92 | 0.0003 | 0.0022 | 0.1615 | 77 |
| 4933428G20Rik | 58996 | 1.38 | 1.16 | 0.0005 | 0.1196 | 0.2460 | 57 |
| 5730410E15Rik | 319613 | 0.23 | 0.82 | 0.0001 | 0.0110 | 0.5222 | 77 |
| 5730449L18Rik | 66637 | 0.47 | 0.60 | 0.0000 | 0.0111 | 0.0049 | 24 |
| 6430604K15Rik | 269997 | 0.58 | 0.94 | 0.0007 | 0.0011 | 0.5278 | 85 |
| 9630015D15Rik | n/a | 1.54 | 1.29 | 0.0010 | 0.0489 | 0.1580 | 47 |
| AA415398 | 433752 | 0.63 | 0.63 | 0.0016 | 0.9293 | 0.0139 | -2 |
| Aars | 234734 | 0.65 | 1.00 | 0.0010 | 0.0001 | 0.9958 | 100 |
| Abcc3 | 76408 | 1.47 | 1.49 | 0.0053 | 0.9287 | 0.0617 | -4 |
| Abi3 | n/a | 0.39 | 0.69 | 0.0004 | 0.0010 | 0.0755 | 49 |
| Acd | 497652 | 0.72 | 0.75 | 0.0021 | 0.4997 | 0.0258 | 11 |
| Acsl1 | 14081 | 1.79 | 1.05 | 0.0006 | 0.0016 | 0.7585 | 93 |
| Actn4 | 60595 | 1.67 | 1.37 | 0.0006 | 0.0280 | 0.0557 | 44 |
| Acvr2b | 11481 | 0.67 | 0.71 | 0.0011 | 0.5634 | 0.0134 | 14 |
| Adnp | 11538 | 0.66 | 0.83 | 0.0001 | 0.0060 | 0.0243 | 51 |
| Adrbk1 | 110355 | 0.63 | 0.66 | 0.0012 | 0.4819 | 0.0145 | 8 |
| Aes | 14797 | 1.60 | 1.35 | 0.0026 | 0.0665 | 0.0787 | 42 |
| Agtr1a | 11607 | 1.33 | 1.05 | 0.0018 | 0.0128 | 0.5043 | 83 |
| Agxt2l1 | 71760 | 2.21 | 1.88 | 0.0028 | 0.4461 | 0.0149 | 27 |
| AI132487 | 104910 | 1.36 | 1.23 | 0.0027 | 0.2532 | 0.0362 | 37 |
| AI316807 | 102032 | 0.67 | 0.82 | 0.0014 | 0.1028 | 0.0978 | 43 |
| AI451557 | 102084 | 0.39 | 1.06 | 0.0001 | 0.0002 | 0.4905 | 110 |
| Akap12 | 83397 | 2.49 | 3.38 | 0.0025 | 0.3813 | 0.0489 | -60 |
| Aldoa | 11674 | 1.62 | 0.94 | 0.0009 | 0.0008 | 0.5243 | 110 |
| Ang | 11727 | 0.66 | 1.04 | 0.0000 | 0.0003 | 0.6759 | 113 |
| Ankzf1 | 52231 | 0.55 | 0.79 | 0.0001 | 0.0000 | 0.0740 | 54 |
| Anxa6 | 11749 | 1.56 | 1.24 | 0.0053 | 0.0607 | 0.2303 | 56 |
| Apcs | 20219 | 0.48 | 1.06 | 0.0003 | 0.0014 | 0.7755 | 111 |
| Apol9b | 71898 | 1.66 | 1.02 | 0.0011 | 0.0032 | 0.9361 | 97 |
| Arfl4 | 66182 | 2.52 | 1.74 | 0.0001 | 0.0161 | 0.0155 | 51 |
| Arhgap18 | 73910 | 1.58 | 1.25 | 0.0011 | 0.0717 | 0.0515 | 56 |
| Arhgap26 | 71302 | 8.47 | 4.03 | 0.0000 | 0.0092 | 0.0525 | 60 |
| Arhgap30 | 226652 | 0.44 | 0.77 | 0.0004 | 0.0006 | 0.1630 | 59 |
| Arhgef18 | 102098 | 0.64 | 0.80 | 0.0031 | 0.0373 | 0.1041 | 46 |
| Arl16 | 70317 | 0.67 | 0.72 | 0.0004 | 0.4244 | 0.0191 | 14 |
| Arl4a | 11861 | 2.47 | 1.44 | 0.0000 | 0.0030 | 0.0171 | 70 |
| Armcx1 | 78248 | 0.39 | 0.62 | 0.0000 | 0.0930 | 0.0154 | 37 |
| Armcx3 | 71703 | 0.66 | 0.87 | 0.0006 | 0.0662 | 0.3248 | 63 |
| Arrdc4 | 66412 | 1.82 | 1.27 | 0.0053 | 0.0793 | 0.1019 | 67 |
| Asb3 | 65257 | 0.51 | 0.86 | 0.0000 | 0.0011 | 0.1047 | 71 |
| Asns | 27053 | 0.15 | 0.68 | 0.0000 | 0.0081 | 0.1654 | 62 |
| Atf4 | 11911 | 0.71 | 0.92 | 0.0001 | 0.0372 | 0.4707 | 72 |
| Atf7ip | 54343 | 0.50 | 0.75 | 0.0004 | 0.0065 | 0.0497 | 50 |
| Atxn1 | 20238 | 1.45 | 0.99 | 0.0079 | 0.0117 | 0.9053 | 102 |
| Axin2 | 12006 | 0.49 | 0.78 | 0.0008 | 0.0508 | 0.2796 | 58 |
| B230312A22Rik | 230088 | 2.52 | 2.74 | 0.0007 | 0.6912 | 0.0068 | -15 |
| B230342M21Rik | 100637 | 0.60 | 0.71 | 0.0011 | 0.3809 | 0.0266 | 28 |
| B4galt3 | 57370 | 0.62 | 0.82 | 0.0000 | 0.0000 | 0.0218 | 54 |
| Bap1 | n/a | 1.55 | 1.37 | 0.0006 | 0.1823 | 0.0063 | 33 |
| BC002199 | 211556 | 0.63 | 0.85 | 0.0001 | 0.0008 | 0.1199 | 61 |
| BC003236 | n/a | 2.12 | 1.43 | 0.0004 | 0.0186 | 0.0452 | 62 |
| BC005537 | 79555 | 1.47 | 1.27 | 0.0000 | 0.0422 | 0.0052 | 43 |
| BC017158 | 233913 | 0.71 | 0.70 | 0.0005 | 0.6648 | 0.0038 | -4 |
| BC017612 | 170748 | 0.61 | 0.70 | 0.0011 | 0.4524 | 0.0414 | 22 |
| BC022224 | 192970 | 1.51 | 1.12 | 0.0006 | 0.0189 | 0.3783 | 77 |
| BC026590 | 230234 | 0.60 | 0.61 | 0.0002 | 0.7012 | 0.0030 | 3 |
| BC031353 | 235493 | 0.55 | 0.65 | 0.0061 | 0.5250 | 0.0711 | 22 |
| BC037034 | 231807 | 0.65 | 0.66 | 0.0005 | 0.8537 | 0.0079 | 3 |
| BC056474 | 414077 | 1.52 | 1.33 | 0.0079 | 0.3276 | 0.0430 | 36 |
| Bcar1 | 12927 | 1.85 | 0.87 | 0.0032 | 0.0031 | 0.2851 | 116 |
| Bcl2l1 | 12048 | 2.21 | 2.65 | 0.0001 | 0.2876 | 0.0035 | -37 |
| Bcl2l13 | 94044 | 1.39 | 1.22 | 0.0003 | 0.0875 | 0.0098 | 43 |
| Bhlhb2 | 20893 | 1.98 | 0.92 | 0.0124 | 0.0150 | 0.4485 | 108 |
| Brf2 | 66653 | 0.63 | 0.76 | 0.0001 | 0.0378 | 0.0030 | 36 |
| Btbd12 | 52864 | 0.53 | 0.71 | 0.0006 | 0.0370 | 0.0483 | 39 |
| Btg1 | 12226 | 1.36 | 1.14 | 0.0031 | 0.1868 | 0.3779 | 61 |
| Bud13 | 215051 | 0.62 | 0.76 | 0.0020 | 0.0921 | 0.0846 | 35 |
| C430004E15Rik | 97031 | 2.41 | 1.27 | 0.0023 | 0.0142 | 0.1165 | 81 |
| Casp1 | 12362 | 0.55 | 0.87 | 0.0012 | 0.0062 | 0.3831 | 71 |
| Ccar1 | n/a | 0.65 | 1.11 | 0.0000 | 0.0000 | 0.2069 | 130 |
| Ccdc120 | 54648 | 2.34 | 1.82 | 0.0136 | 0.3365 | 0.0248 | 39 |
| Ccdc130 | 67736 | 0.41 | 0.69 | 0.0000 | 0.0009 | 0.0025 | 47 |
| Ccdc131 | 216345 | 0.46 | 0.82 | 0.0000 | 0.0028 | 0.1567 | 66 |
| Ccdc134 | 76457 | 0.35 | 0.66 | 0.0000 | 0.0425 | 0.0969 | 48 |
| Ccnl1 | 56706 | 1.90 | 1.33 | 0.0029 | 0.0657 | 0.0399 | 63 |
| Ccr5 | 12774 | 0.17 | 0.46 | 0.0000 | 0.0084 | 0.0126 | 35 |
| Cd14 | 12475 | 3.06 | 3.93 | 0.0003 | 0.4732 | 0.0484 | -42 |
| Cd83 | 12522 | 6.86 | 1.96 | 0.0000 | 0.0001 | 0.2373 | 84 |
| Cdc42bpb | 217866 | 1.54 | 1.11 | 0.0028 | 0.0357 | 0.3891 | 79 |
| Cdc42ep4 | 56699 | 1.50 | 1.23 | 0.0020 | 0.0161 | 0.1802 | 55 |
| Cdc42ep5 | 58804 | 4.63 | 1.30 | 0.0048 | 0.0161 | 0.4133 | 92 |
| Cdkn1a | 12575 | 3.32 | 3.14 | 0.0004 | 0.8069 | 0.0119 | 8 |
| Cebpb | 12608 | 1.89 | 2.07 | 0.0001 | 0.1576 | 0.0008 | -19 |
| Chac1 | 69065 | 0.24 | 0.34 | 0.0000 | 0.0077 | 0.0011 | 14 |
| Chchd8 | 68185 | 0.43 | 0.64 | 0.0001 | 0.0091 | 0.0165 | 36 |
| Chkb | 12651 | 1.81 | 1.26 | 0.0005 | 0.0151 | 0.0851 | 67 |
| Chordc1 | 66917 | 0.61 | 0.83 | 0.0000 | 0.0364 | 0.1206 | 56 |
| Chrd | 12667 | 0.46 | 0.61 | 0.0001 | 0.0106 | 0.0110 | 28 |
| Cirbp | 12696 | 2.23 | 1.39 | 0.0000 | 0.0068 | 0.1263 | 68 |
| Clk4 | 12750 | 0.87 | 1.12 | 0.0166 | 0.0001 | 0.0306 | 192 |
| Clp1 | 98985 | 0.53 | 0.93 | 0.0000 | 0.0003 | 0.4819 | 84 |
| Cml2 | 93673 | 0.52 | 0.84 | 0.0000 | 0.0028 | 0.0324 | 67 |
| Cpeb2 | 231207 | 2.91 | 0.58 | 0.0045 | 0.0023 | 0.0231 | 122 |
| Cpne8 | 66871 | 0.22 | 1.23 | 0.0001 | 0.0036 | 0.5340 | 130 |
| Crcp | 12909 | 1.67 | 2.02 | 0.0004 | 0.0137 | 0.0001 | -53 |
| Creb3l3 | 208677 | 1.44 | 1.24 | 0.0000 | 0.0849 | 0.0900 | 44 |
| Cry2 | 12953 | 1.62 | 1.75 | 0.0043 | 0.6362 | 0.0111 | -20 |
| Cs | 12974 | 1.40 | 1.26 | 0.0000 | 0.0430 | 0.0050 | 35 |
| Csnk1d | 104318 | 1.61 | 1.02 | 0.0010 | 0.0026 | 0.7489 | 96 |
| Csnk1e | 27373 | 1.93 | 1.30 | 0.0001 | 0.0055 | 0.0052 | 68 |
| Ctsl | 13039 | 2.03 | 2.75 | 0.0161 | 0.2423 | 0.0078 | -70 |
| Cttnbp2nl | 80281 | 2.29 | 1.48 | 0.0014 | 0.0392 | 0.1084 | 63 |
| Cxcl1 | 14825 | 0.38 | 0.96 | 0.0013 | 0.0130 | 0.8186 | 94 |
| Cxcl4 | 56744 | 1.66 | 2.20 | 0.0014 | 0.4079 | 0.1316 | -82 |
| Cyp4f15 | 106648 | 1.38 | 1.50 | 0.0094 | 0.4543 | 0.0121 | -33 |
| D030074E01Rik | 75964 | 2.31 | 1.04 | 0.0018 | 0.0035 | 0.7853 | 97 |
| D11Wsu47e | 276852 | 0.32 | 0.61 | 0.0000 | 0.0001 | 0.0089 | 43 |
| D15Ertd682e | 71919 | 0.51 | 0.89 | 0.0001 | 0.0006 | 0.3356 | 79 |
| D6Wsu163e | 28040 | 0.50 | 0.72 | 0.0000 | 0.0026 | 0.0012 | 44 |
| D930015E06Rik | 229473 | 2.61 | 0.89 | 0.0046 | 0.0061 | 0.6624 | 107 |
| Dact2 | 240025 | 3.25 | 1.23 | 0.0072 | 0.0329 | 0.5637 | 90 |
| Dgat2 | 67800 | 1.37 | 1.18 | 0.0056 | 0.0982 | 0.1029 | 53 |
| Dhx30 | 72831 | 0.70 | 0.80 | 0.0008 | 0.0112 | 0.0343 | 34 |
| Dnajb10 | 56812 | 0.63 | 0.72 | 0.0002 | 0.1252 | 0.0208 | 26 |
| Dnajb9 | 27362 | 0.46 | 0.95 | 0.0000 | 0.0045 | 0.7878 | 91 |
| Dnmbp | 71972 | 6.94 | 3.64 | 0.0015 | 0.0656 | 0.0019 | 56 |
| Dok3 | 27261 | 0.48 | 0.84 | 0.0001 | 0.0028 | 0.1955 | 70 |
| Dom3z | 112403 | 0.73 | 0.84 | 0.0002 | 0.0461 | 0.0476 | 40 |
| Dot1l | 208266 | 2.55 | 1.34 | 0.0002 | 0.0056 | 0.1598 | 78 |
| Dph2 | 67728 | 0.52 | 0.97 | 0.0000 | 0.0000 | 0.7430 | 94 |
| Dpp8 | 74388 | 0.64 | 0.84 | 0.0005 | 0.0012 | 0.1007 | 56 |
| Dusp11 | 72102 | 0.72 | 0.97 | 0.0001 | 0.0001 | 0.5792 | 89 |
| Dusp16 | 70686 | 1.76 | 1.59 | 0.0000 | 0.1980 | 0.0031 | 22 |
| Dyrk2 | 69181 | 2.45 | 1.16 | 0.0003 | 0.0025 | 0.4973 | 89 |
| E130012A19Rik | 103551 | 2.07 | 2.12 | 0.0013 | 0.8838 | 0.0003 | -5 |
| Eef1e1 | 66143 | 0.57 | 1.14 | 0.0003 | 0.0001 | 0.0653 | 133 |
| EG622320 | 622320 | 0.37 | 0.61 | 0.0001 | 0.0672 | 0.0175 | 38 |
| Eif2ak2 | 19106 | 0.51 | 0.80 | 0.0000 | 0.0017 | 0.0733 | 59 |
| Eif2b4 | 13667 | 0.73 | 0.95 | 0.0010 | 0.0005 | 0.5840 | 82 |
| Eif4ebp1 | 13685 | 0.60 | 0.76 | 0.0002 | 0.0897 | 0.0181 | 40 |
| Eif4ebp2 | 13688 | 1.44 | 0.96 | 0.0034 | 0.0006 | 0.7741 | 109 |
| Ell | 13716 | 0.65 | 0.92 | 0.0023 | 0.0076 | 0.4978 | 78 |
| Eng | 13805 | 1.47 | 1.20 | 0.0034 | 0.1104 | 0.0941 | 57 |
| ENSMUSG00000053178 | 208595 | 0.44 | 0.64 | 0.0001 | 0.0127 | 0.0148 | 36 |
| Eps8l2 | 98845 | 1.58 | 0.63 | 0.0012 | 0.0000 | 0.0005 | 164 |
| Erdr1 | 170942 | 1.82 | 1.59 | 0.0045 | 0.4729 | 0.0544 | 28 |
| Es22 | 13897 | 1.47 | 1.26 | 0.0048 | 0.1533 | 0.0395 | 44 |
| Ets2 | 23872 | 1.73 | 1.58 | 0.0016 | 0.6055 | 0.0553 | 21 |
| Ext1 | 14042 | 1.57 | 1.70 | 0.0004 | 0.4606 | 0.0054 | -23 |
| Extl1 | 56219 | 0.44 | 0.53 | 0.0011 | 0.2730 | 0.0130 | 16 |
| F11r | 16456 | 1.51 | 0.89 | 0.0001 | 0.0000 | 0.1863 | 122 |
| Fadd | 14082 | 0.67 | 0.61 | 0.0001 | 0.1722 | 0.0007 | -18 |
| Farsb | 23874 | 0.74 | 0.96 | 0.0002 | 0.0031 | 0.5957 | 84 |
| Fas | 14102 | 0.69 | 1.03 | 0.0001 | 0.0013 | 0.7984 | 109 |
| Fastkd5 | 380601 | 0.56 | 1.05 | 0.0000 | 0.0000 | 0.5685 | 112 |
| Fbf1 | 217335 | 4.50 | 4.73 | 0.0020 | 0.8831 | 0.0285 | -7 |
| Fbxo21 | 231670 | 1.62 | 0.89 | 0.0001 | 0.0001 | 0.3711 | 118 |
| Fbxo25 | 66822 | 0.64 | 0.64 | 0.0019 | 0.9652 | 0.0170 | 1 |
| Fbxo30 | 71865 | 0.59 | 0.91 | 0.0004 | 0.0105 | 0.4410 | 78 |
| Fbxo34 | 78938 | 1.73 | 1.65 | 0.0002 | 0.6224 | 0.0005 | 11 |
| Fcgr3 | 14131 | 0.66 | 1.05 | 0.0002 | 0.0003 | 0.6106 | 114 |
| Fem1b | 14155 | 0.65 | 0.92 | 0.0000 | 0.0014 | 0.3568 | 78 |
| Fermt2 | 218952 | 0.54 | 0.56 | 0.0020 | 0.8192 | 0.0158 | 3 |
| Fgf21 | 56636 | 0.03 | 0.12 | 0.0000 | 0.0306 | 0.0007 | 9 |
| Fgfrl1 | 116701 | 1.54 | 1.02 | 0.0007 | 0.0065 | 0.8933 | 96 |
| Fgl1 | 234199 | 0.65 | 1.09 | 0.0004 | 0.0000 | 0.3263 | 125 |
| Fkbp4 | 14228 | 1.69 | 0.96 | 0.0007 | 0.0005 | 0.7772 | 105 |
| Flcn | 216805 | 0.36 | 0.60 | 0.0000 | 0.0004 | 0.0103 | 38 |
| Flot1 | 14251 | 0.50 | 0.99 | 0.0000 | 0.0000 | 0.9552 | 99 |
| Flvcr2 | 217721 | 2.00 | 0.64 | 0.0043 | 0.0010 | 0.0493 | 136 |
| Fndc3b | 72007 | 0.55 | 1.32 | 0.0004 | 0.0004 | 0.1591 | 171 |
| Foxa3 | 15377 | 0.74 | 0.70 | 0.0024 | 0.5906 | 0.0185 | -17 |
| Foxp1 | 108655 | 2.90 | 1.63 | 0.0000 | 0.0012 | 0.0213 | 67 |
| Fpr2 | 14289 | 0.53 | 1.52 | 0.0003 | 0.0460 | 0.3430 | 211 |
| Fst | 14313 | 5.34 | 4.57 | 0.0016 | 0.6367 | 0.0214 | 18 |
| G0s2 | 14373 | 0.29 | 0.16 | 0.0004 | 0.0745 | 0.0011 | -18 |
| G6pc | 14377 | 2.64 | 1.31 | 0.0004 | 0.0066 | 0.2609 | 81 |
| Gadd45b | 17873 | 10.25 | 3.81 | 0.0001 | 0.0060 | 0.0159 | 70 |
| Gamt | 14431 | 0.68 | 0.70 | 0.0027 | 0.7534 | 0.0256 | 6 |
| Gars | 353172 | 0.65 | 0.85 | 0.0001 | 0.0186 | 0.0671 | 58 |
| Gbl | 56716 | 0.64 | 0.70 | 0.0003 | 0.1613 | 0.0118 | 17 |
| Gck | 103988 | 2.78 | 1.38 | 0.0008 | 0.0070 | 0.0700 | 78 |
| Gfod2 | 70575 | 0.36 | 0.77 | 0.0001 | 0.0036 | 0.1197 | 64 |
| Gimap9 | 317758 | 0.48 | 1.19 | 0.0013 | 0.0001 | 0.1981 | 137 |
| Gja4 | 14612 | 2.49 | 2.15 | 0.0003 | 0.4454 | 0.0235 | 23 |
| Gna12 | 14673 | 2.70 | 1.45 | 0.0000 | 0.0010 | 0.1579 | 74 |
| Gnat1 | 14685 | 0.42 | 1.18 | 0.0010 | 0.0002 | 0.2488 | 131 |
| Golga1 | 76899 | 0.52 | 0.68 | 0.0000 | 0.0547 | 0.0024 | 33 |
| Golga3 | 269682 | 0.58 | 0.98 | 0.0000 | 0.0000 | 0.8176 | 96 |
| Golph3l | 229593 | 0.44 | 0.80 | 0.0001 | 0.0031 | 0.2321 | 64 |
| Gpatch2 | 67769 | 0.61 | 0.95 | 0.0000 | 0.0002 | 0.2483 | 88 |
| Gtf2e1 | 74197 | 0.62 | 1.03 | 0.0003 | 0.0001 | 0.7633 | 109 |
| Gtf2h3 | 209357 | 0.62 | 0.70 | 0.0002 | 0.0596 | 0.0076 | 20 |
| H3f3b | 15081 | 2.06 | 1.95 | 0.0000 | 0.5370 | 0.0001 | 10 |
| Hax1 | 23897 | 0.69 | 0.86 | 0.0000 | 0.0038 | 0.0354 | 55 |
| Hdgfrp2 | 15193 | 0.74 | 0.74 | 0.0006 | 0.8648 | 0.0030 | -2 |
| Hdhd3 | 72748 | 0.69 | 0.57 | 0.0020 | 0.1943 | 0.0001 | -39 |
| Heatr1 | 217995 | 0.57 | 0.98 | 0.0001 | 0.0013 | 0.8485 | 94 |
| Herpud1 | 64209 | 1.43 | 1.29 | 0.0014 | 0.1514 | 0.0597 | 34 |
| Hhex | 15242 | 0.40 | 0.73 | 0.0050 | 0.1665 | 0.2822 | 55 |
| Hic2 | 58180 | 3.58 | 1.91 | 0.0005 | 0.0237 | 0.0678 | 65 |
| Hist1h1c | 50708 | 2.92 | 1.00 | 0.0000 | 0.0001 | 0.9855 | 100 |
| Hist2h3b | 319154 | 2.02 | 1.85 | 0.0001 | 0.4766 | 0.0007 | 16 |
| Hist2h3c1 | 15077 | 2.02 | 1.81 | 0.0000 | 0.2801 | 0.0002 | 20 |
| Hnrpl | 15388 | 1.36 | 1.26 | 0.0001 | 0.1851 | 0.0301 | 28 |
| Iars | 105148 | 0.65 | 0.97 | 0.0001 | 0.0004 | 0.7385 | 93 |
| Ibtk | n/a | 0.68 | 0.88 | 0.0025 | 0.0094 | 0.3302 | 64 |
| Icam1 | 15894 | 0.58 | 1.09 | 0.0000 | 0.0498 | 0.7575 | 121 |
| Id2 | 15902 | 0.49 | 0.62 | 0.0008 | 0.0503 | 0.0251 | 26 |
| Ifi47 | 15953 | 0.43 | 0.75 | 0.0008 | 0.0032 | 0.1946 | 56 |
| Igfbp2 | 16008 | 1.53 | 1.56 | 0.0035 | 0.8322 | 0.0001 | -7 |
| Igtp | 16145 | 0.66 | 0.79 | 0.0013 | 0.1542 | 0.0966 | 39 |
| Iigp2 | 54396 | 0.56 | 0.81 | 0.0006 | 0.0305 | 0.2065 | 57 |
| Il13ra1 | 16164 | 0.65 | 1.12 | 0.0016 | 0.0012 | 0.4318 | 134 |
| Il15ra | 16169 | 0.51 | 0.92 | 0.0000 | 0.0018 | 0.4202 | 84 |
| Il16 | 16170 | 0.24 | 0.58 | 0.0015 | 0.0530 | 0.1856 | 45 |
| Impact | 16210 | 1.42 | 1.36 | 0.0026 | 0.6648 | 0.0001 | 13 |
| Inhbe | 16326 | 0.10 | 0.27 | 0.0000 | 0.0010 | 0.0004 | 19 |
| Insc | 233752 | 0.53 | 1.86 | 0.0016 | 0.0001 | 0.0094 | 285 |
| Insig1 | 231070 | 1.90 | 1.08 | 0.0016 | 0.0057 | 0.6093 | 91 |
| Ipmk | 69718 | 1.53 | 1.04 | 0.0087 | 0.0383 | 0.7879 | 92 |
| Irak3 | 73914 | 0.43 | 1.38 | 0.0003 | 0.0338 | 0.4535 | 166 |
| Irf1 | 16362 | 0.50 | 0.57 | 0.0001 | 0.2113 | 0.0022 | 15 |
| Irf2bp1 | 272359 | 0.59 | 0.60 | 0.0009 | 0.8987 | 0.0079 | 2 |
| Itgb5 | 16419 | 1.33 | 0.88 | 0.0010 | 0.0000 | 0.2405 | 137 |
| Jdp2 | 81703 | 3.82 | 2.13 | 0.0007 | 0.0191 | 0.0470 | 60 |
| Jun | 16476 | 1.69 | 1.81 | 0.0009 | 0.5974 | 0.0096 | -17 |
| Jund1 | 16478 | 3.17 | 1.50 | 0.0018 | 0.0129 | 0.1177 | 77 |
| Kctd2 | 70382 | 0.47 | 0.45 | 0.0014 | 0.7387 | 0.0102 | -3 |
| Klb | 83379 | 1.76 | 1.09 | 0.0001 | 0.0020 | 0.5108 | 89 |
| Klf13 | 50794 | 1.56 | 1.82 | 0.0060 | 0.1901 | 0.0034 | -47 |
| Klf2 | 16598 | 1.97 | 2.07 | 0.0052 | 0.7692 | 0.0055 | -10 |
| Klf7 | 93691 | 1.47 | 1.13 | 0.0030 | 0.0412 | 0.4411 | 73 |
| Krcc1 | 57896 | 0.74 | 0.80 | 0.0001 | 0.1696 | 0.0083 | 24 |
| Krt10 | 16661 | 1.43 | 1.08 | 0.0006 | 0.0124 | 0.3558 | 81 |
| Krt23 | 94179 | 1.84 | 0.54 | 0.0058 | 0.0005 | 0.0206 | 155 |
| Krt8 | 16691 | 2.13 | 1.16 | 0.0055 | 0.0160 | 0.5518 | 86 |
| Lars | 107045 | 0.63 | 1.11 | 0.0000 | 0.0011 | 0.4374 | 129 |
| Lcn2 | 16819 | 0.20 | 2.68 | 0.0028 | 0.0000 | 0.0007 | 311 |
| Ldlr | 16835 | 4.50 | 1.30 | 0.0000 | 0.0001 | 0.4476 | 92 |
| Lhfpl2 | 218454 | 0.61 | 0.48 | 0.0049 | 0.2992 | 0.0011 | -34 |
| Litaf | 56722 | 0.62 | 0.98 | 0.0001 | 0.0010 | 0.8448 | 94 |
| Lmo4 | 16911 | 0.69 | 0.95 | 0.0045 | 0.0697 | 0.7649 | 84 |
| Lnx2 | 140887 | 2.20 | 1.38 | 0.0114 | 0.0987 | 0.0128 | 68 |
| LOC100041103 | 100041103 | 1.37 | 1.03 | 0.0015 | 0.0076 | 0.7494 | 92 |
| LOC100044862 | 100044862 | 1.41 | 1.26 | 0.0000 | 0.0206 | 0.0015 | 38 |
| LOC100045343 | n/a | 0.18 | 0.54 | 0.0000 | 0.0002 | 0.0305 | 44 |
| LOC100045963 | 100045963 | 1.55 | 1.26 | 0.0001 | 0.0296 | 0.0879 | 52 |
| LOC100046406 | 100046406 | 0.44 | 0.90 | 0.0006 | 0.0040 | 0.4710 | 81 |
| LOC100046891 | n/a | 0.50 | 0.75 | 0.0000 | 0.0003 | 0.0145 | 51 |
| LOC100047200 | 100047200 | 0.30 | 0.49 | 0.0003 | 0.0079 | 0.0167 | 26 |
| LOC100047427 | 100047427 | 4.22 | 1.06 | 0.0001 | 0.0003 | 0.6144 | 98 |
| LOC100047707 | 100047707 | 1.37 | 1.27 | 0.0004 | 0.1873 | 0.0366 | 27 |
| LOC100047762 | 100047762 | 2.33 | 3.10 | 0.0004 | 0.0378 | 0.0000 | -57 |
| LOC100047834 | 100047834 | 0.69 | 0.79 | 0.0039 | 0.1913 | 0.0675 | 32 |
| LOC100047911 | 100047911 | 0.49 | 0.59 | 0.0000 | 0.2808 | 0.0037 | 20 |
| LOC100047934 | 100047934 | 0.27 | 0.69 | 0.0001 | 0.0063 | 0.1776 | 58 |
| LOC100048105 | 100048105 | 1.46 | 1.06 | 0.0080 | 0.0372 | 0.4991 | 87 |
| LOC216443 | n/a | 0.58 | 0.72 | 0.0001 | 0.0307 | 0.0054 | 34 |
| LOC381302 | n/a | 0.43 | 0.57 | 0.0000 | 0.0662 | 0.0035 | 25 |
| LOC382010 | n/a | 0.57 | 0.80 | 0.0006 | 0.0110 | 0.1617 | 55 |
| Loh11cr2a | 67776 | 0.71 | 0.86 | 0.0031 | 0.0785 | 0.1872 | 50 |
| Lpgat1 | 226856 | 0.68 | 0.87 | 0.0056 | 0.0375 | 0.1943 | 61 |
| Lpin2 | n/a | 3.22 | 1.70 | 0.0001 | 0.0069 | 0.1290 | 69 |
| Lrfn3 | 233067 | 2.33 | 0.84 | 0.0073 | 0.0071 | 0.2294 | 112 |
| Lrg1 | 76905 | 0.56 | 0.98 | 0.0000 | 0.0001 | 0.6464 | 96 |
| Lrig1 | 16206 | 0.50 | 0.72 | 0.0001 | 0.0051 | 0.0146 | 44 |
| Lrrc29 | 234684 | 0.17 | 0.60 | 0.0000 | 0.0001 | 0.0086 | 52 |
| Lrrc3 | 237387 | 1.35 | 0.88 | 0.0022 | 0.0003 | 0.2183 | 134 |
| Lypla3 | 192654 | 0.63 | 0.58 | 0.0001 | 0.1976 | 0.0006 | -12 |
| Lysmd4 | 75099 | 0.65 | 1.19 | 0.0011 | 0.0003 | 0.1053 | 155 |
| Lztr1 | 66863 | 0.71 | 0.74 | 0.0016 | 0.4831 | 0.0306 | 12 |
| Map3k1 | 26401 | 0.50 | 1.03 | 0.0019 | 0.0037 | 0.8088 | 105 |
| Mapk6 | 50772 | 1.62 | 1.56 | 0.0001 | 0.6830 | 0.0008 | 9 |
| Mapkapk2 | 17164 | 2.30 | 1.92 | 0.0015 | 0.3374 | 0.0024 | 29 |
| Mbc2 | 23943 | 0.67 | 0.72 | 0.0007 | 0.3409 | 0.0064 | 16 |
| Mbd3 | 17192 | 1.57 | 1.22 | 0.0007 | 0.0120 | 0.0675 | 61 |
| Mboat5 | 14792 | 1.52 | 0.85 | 0.0000 | 0.0000 | 0.0953 | 128 |
| Mcl1 | 17210 | 0.70 | 0.92 | 0.0017 | 0.0007 | 0.3423 | 72 |
| Med31 | 67279 | 0.59 | 0.86 | 0.0000 | 0.0010 | 0.1385 | 66 |
| Meis1 | 17268 | 0.57 | 0.62 | 0.0064 | 0.7070 | 0.0076 | 12 |
| Mettl3 | 56335 | 0.47 | 0.61 | 0.0001 | 0.0586 | 0.0086 | 26 |
| Mettl6 | 67011 | 0.43 | 1.02 | 0.0002 | 0.0000 | 0.8534 | 104 |
| Mknk1 | 17346 | 0.63 | 0.84 | 0.0001 | 0.0140 | 0.0579 | 56 |
| Mknk2 | 17347 | 2.02 | 1.78 | 0.0002 | 0.2976 | 0.0133 | 24 |
| Mmp15 | 17388 | 1.96 | 1.01 | 0.0021 | 0.0138 | 0.9706 | 99 |
| Mphosph6 | 68533 | 1.65 | 1.65 | 0.0065 | 0.9927 | 0.0045 | 0 |
| Mrap | 77037 | 1.43 | 1.90 | 0.0014 | 0.0439 | 0.0024 | -113 |
| Mreg | 381269 | 1.41 | 0.86 | 0.0028 | 0.0001 | 0.2647 | 133 |
| Mrm1 | 217038 | 0.45 | 0.61 | 0.0004 | 0.0586 | 0.0371 | 29 |
| Mrpl50 | 28028 | 0.73 | 0.78 | 0.0005 | 0.0408 | 0.0125 | 16 |
| Mtap7d1 | 245877 | 0.57 | 0.87 | 0.0000 | 0.0000 | 0.1030 | 70 |
| Mterf | 545725 | 0.42 | 0.70 | 0.0000 | 0.0005 | 0.0310 | 48 |
| Mthfd2 | 17768 | 0.16 | 0.67 | 0.0000 | 0.0009 | 0.0885 | 61 |
| Mtmr3 | 74302 | 2.08 | 1.42 | 0.0000 | 0.0041 | 0.0932 | 61 |
| Mtnr1a | 17773 | 2.00 | 0.88 | 0.0020 | 0.0004 | 0.6389 | 112 |
| Myadm | 50918 | 1.80 | 1.45 | 0.0003 | 0.1614 | 0.0717 | 44 |
| Mycl1 | 16918 | 1.87 | 0.84 | 0.0020 | 0.0007 | 0.3935 | 119 |
| Myd88 | 17874 | 0.54 | 1.15 | 0.0000 | 0.0002 | 0.2716 | 132 |
| Narf | 67608 | 0.55 | 1.04 | 0.0005 | 0.0001 | 0.7007 | 109 |
| Ncbp2 | 68092 | 0.51 | 0.64 | 0.0003 | 0.2536 | 0.0343 | 28 |
| Ncf4 | 17972 | 0.32 | 0.85 | 0.0011 | 0.0021 | 0.5671 | 78 |
| Ncoa5 | 228869 | 0.57 | 0.81 | 0.0000 | 0.0002 | 0.0403 | 55 |
| Ndrg1 | 17988 | 2.40 | 1.52 | 0.0009 | 0.0461 | 0.0537 | 63 |
| Ndrl | n/a | 2.27 | 1.61 | 0.0040 | 0.1439 | 0.0141 | 52 |
| Nedd4l | 83814 | 0.68 | 1.12 | 0.0017 | 0.0014 | 0.3578 | 137 |
| Nfkbia | 18035 | 1.98 | 1.87 | 0.0011 | 0.6242 | 0.0199 | 11 |
| Nfkbib | n/a | 3.26 | 2.62 | 0.0000 | 0.2077 | 0.0027 | 28 |
| Nmd3 | 97112 | 1.53 | 1.42 | 0.0002 | 0.4758 | 0.0156 | 20 |
| Nol10 | 217431 | 0.69 | 1.14 | 0.0007 | 0.0001 | 0.1108 | 145 |
| Nola1 | 68147 | 0.67 | 0.91 | 0.0013 | 0.0129 | 0.4790 | 74 |
| Nos3 | 18127 | 0.41 | 1.12 | 0.0016 | 0.0001 | 0.5277 | 120 |
| Nploc4 | 217365 | 1.51 | 1.62 | 0.0004 | 0.3253 | 0.0006 | -23 |
| Nr1d1 | 217166 | 4.96 | 1.17 | 0.0001 | 0.0006 | 0.4059 | 96 |
| Nr1h4 | 20186 | 0.51 | 0.62 | 0.0000 | 0.0211 | 0.0000 | 23 |
| Nr2f6 | 13864 | 1.48 | 1.01 | 0.0026 | 0.0057 | 0.8921 | 98 |
| Nsmce1 | 67711 | 0.69 | 0.75 | 0.0003 | 0.1461 | 0.0106 | 22 |
| Nudt4 | 71207 | 1.38 | 1.21 | 0.0003 | 0.0955 | 0.0320 | 44 |
| Nup62 | 18226 | 1.40 | 1.25 | 0.0072 | 0.2290 | 0.0371 | 37 |
| Nupl2 | 231042 | 0.41 | 0.69 | 0.0000 | 0.0003 | 0.0018 | 49 |
| Obfc2a | 109019 | 0.60 | 0.74 | 0.0024 | 0.2133 | 0.0327 | 34 |
| Optn | 71648 | 1.35 | 1.08 | 0.0002 | 0.0074 | 0.2842 | 77 |
| Oraov1 | 72284 | 0.40 | 0.49 | 0.0003 | 0.2439 | 0.0095 | 14 |
| Orm1 | 18405 | 0.70 | 1.43 | 0.0076 | 0.0000 | 0.0021 | 246 |
| Orm2 | n/a | 0.41 | 1.79 | 0.0020 | 0.0004 | 0.0543 | 233 |
| Osmr | 18414 | 0.60 | 1.43 | 0.0027 | 0.0529 | 0.3622 | 209 |
| Ovca2 | 246257 | 0.64 | 0.71 | 0.0013 | 0.1092 | 0.0310 | 19 |
| P2ry13 | 74191 | 0.22 | 0.51 | 0.0000 | 0.0071 | 0.0029 | 37 |
| P2ry6 | 233571 | 0.42 | 0.60 | 0.0004 | 0.0041 | 0.0292 | 31 |
| Pah | 18478 | 1.52 | 1.51 | 0.0113 | 0.9595 | 0.0268 | 2 |
| Parp16 | 214424 | 1.52 | 1.14 | 0.0021 | 0.0954 | 0.5736 | 73 |
| Pdcl | 67466 | 0.63 | 0.91 | 0.0006 | 0.0027 | 0.4792 | 76 |
| Pelo | 105083 | 0.64 | 0.91 | 0.0000 | 0.0017 | 0.3583 | 75 |
| Per1 | 18626 | 2.67 | 2.46 | 0.0019 | 0.6952 | 0.0133 | 13 |
| Pgpep1 | 66522 | 1.54 | 0.74 | 0.0008 | 0.0000 | 0.0509 | 148 |
| Pgs1 | 74451 | 0.67 | 0.97 | 0.0001 | 0.0019 | 0.7464 | 91 |
| Phactr4 | 100169 | 0.74 | 0.90 | 0.0002 | 0.0051 | 0.0827 | 63 |
| Phf13 | 230936 | 1.70 | 1.05 | 0.0002 | 0.0007 | 0.6119 | 93 |
| Phospho2 | 73373 | 0.73 | 0.93 | 0.0000 | 0.0065 | 0.2560 | 73 |
| Pim3 | n/a | 2.15 | 1.58 | 0.0107 | 0.1822 | 0.0221 | 49 |
| Pla2g6 | 53357 | 1.68 | 1.30 | 0.0005 | 0.0410 | 0.0290 | 55 |
| Plekhf2 | 71801 | 1.51 | 1.19 | 0.0001 | 0.0103 | 0.0177 | 63 |
| Plekhg5 | 269608 | 2.95 | 1.13 | 0.0006 | 0.0044 | 0.6486 | 93 |
| Plrg1 | 53317 | 0.59 | 1.05 | 0.0000 | 0.0000 | 0.5487 | 113 |
| Pls3 | 102866 | 0.63 | 0.93 | 0.0028 | 0.0086 | 0.6068 | 81 |
| Pole4 | 66979 | 0.64 | 1.24 | 0.0034 | 0.0001 | 0.0659 | 169 |
| Ppargc1a | 19017 | 2.47 | 2.18 | 0.0059 | 0.5718 | 0.0104 | 20 |
| Ppargc1b | 170826 | 6.78 | 2.16 | 0.0000 | 0.0011 | 0.0509 | 80 |
| Ppl | 19041 | 2.06 | 1.45 | 0.0004 | 0.0551 | 0.0491 | 58 |
| Ppp1r10 | 52040 | 0.33 | 0.89 | 0.0001 | 0.0001 | 0.3405 | 83 |
| Ppp2r2d | 52432 | 1.60 | 1.37 | 0.0001 | 0.0849 | 0.0014 | 39 |
| Prdm4 | 72843 | 0.61 | 1.08 | 0.0001 | 0.0001 | 0.3931 | 119 |
| Prmt3 | 71974 | 0.73 | 0.78 | 0.0022 | 0.3400 | 0.0132 | 20 |
| Prox1 | 19130 | 0.56 | 0.71 | 0.0000 | 0.0915 | 0.0019 | 35 |
| Prtn3 | 19152 | 0.26 | 0.31 | 0.0019 | 0.2944 | 0.0174 | 7 |
| Psat1 | 107272 | 0.29 | 0.85 | 0.0010 | 0.0265 | 0.4430 | 79 |
| Pscd3 | 19159 | 1.56 | 1.58 | 0.0002 | 0.9339 | 0.0244 | -3 |
| Pscdbp | 227929 | 3.79 | 2.23 | 0.0000 | 0.0253 | 0.0318 | 56 |
| Ptplad1 | 57874 | 0.70 | 0.81 | 0.0001 | 0.0260 | 0.0130 | 37 |
| Ptpn1 | 19246 | 0.49 | 0.79 | 0.0001 | 0.0125 | 0.1403 | 58 |
| Pus7l | 78895 | 0.35 | 0.83 | 0.0000 | 0.0000 | 0.0352 | 74 |
| Pvr | 52118 | 2.40 | 2.12 | 0.0007 | 0.4515 | 0.0002 | 20 |
| Pygo2 | n/a | 0.61 | 0.79 | 0.0000 | 0.0059 | 0.0293 | 45 |
| Qrsl1 | 76563 | 0.63 | 0.70 | 0.0025 | 0.1078 | 0.0323 | 18 |
| Rab40c | 224624 | 0.73 | 0.84 | 0.0056 | 0.1304 | 0.0883 | 40 |
| Rab43 | 69834 | 1.77 | 1.30 | 0.0001 | 0.0283 | 0.1327 | 61 |
| Rabgef1 | 56715 | 1.57 | 0.97 | 0.0035 | 0.0045 | 0.6693 | 104 |
| Rad1 | 19355 | 0.33 | 0.86 | 0.0002 | 0.0003 | 0.4313 | 79 |
| Rars | 104458 | 0.74 | 1.12 | 0.0001 | 0.0038 | 0.3418 | 147 |
| Rars2 | 109093 | 0.62 | 0.87 | 0.0000 | 0.0003 | 0.0030 | 66 |
| Rassf4 | 213391 | 0.43 | 0.64 | 0.0001 | 0.0037 | 0.0128 | 36 |
| Rbm5 | 83486 | 0.46 | 0.71 | 0.0000 | 0.0001 | 0.0067 | 46 |
| Rbms1 | 56878 | 1.35 | 1.18 | 0.0006 | 0.0526 | 0.0283 | 49 |
| Rce1 | 19671 | 1.55 | 1.16 | 0.0028 | 0.0367 | 0.1723 | 72 |
| Rcl1 | 59028 | 1.62 | 1.40 | 0.0000 | 0.0782 | 0.0100 | 35 |
| Reep3 | 28193 | 1.35 | 1.48 | 0.0010 | 0.2282 | 0.0010 | -37 |
| Rell1 | 100532 | 2.92 | 1.11 | 0.0001 | 0.0004 | 0.5938 | 94 |
| Rffl | 67338 | 1.44 | 1.51 | 0.0009 | 0.5373 | 0.0014 | -15 |
| Rg9mtd1 | 52575 | 0.73 | 1.20 | 0.0003 | 0.0001 | 0.0294 | 175 |
| Rgs1 | 50778 | 9.29 | 4.51 | 0.0001 | 0.0165 | 0.0085 | 58 |
| Rhbdd1 | 76867 | 0.70 | 1.23 | 0.0033 | 0.0003 | 0.0949 | 177 |
| Rhob | 11852 | 1.65 | 1.64 | 0.0007 | 0.9427 | 0.0195 | 2 |
| Rhou | 69581 | 1.46 | 1.33 | 0.0006 | 0.3372 | 0.0146 | 29 |
| Ribc1 | 66611 | 0.31 | 0.53 | 0.0001 | 0.0001 | 0.0142 | 32 |
| Ripk4 | 72388 | 2.21 | 1.76 | 0.0000 | 0.0112 | 0.0024 | 38 |
| Rnase4 | 58809 | 0.72 | 0.98 | 0.0000 | 0.0001 | 0.5132 | 91 |
| Rnf113a1 | 69942 | 0.54 | 0.89 | 0.0003 | 0.0002 | 0.3876 | 76 |
| Rnf135 | 71956 | 0.50 | 0.56 | 0.0000 | 0.0677 | 0.0009 | 13 |
| Rnf185 | 193670 | 0.57 | 0.85 | 0.0000 | 0.0003 | 0.1032 | 65 |
| Rogdi | 66049 | 1.44 | 1.14 | 0.0011 | 0.0150 | 0.2098 | 68 |
| Rora | 19883 | 1.62 | 1.25 | 0.0011 | 0.0164 | 0.0928 | 59 |
| rp9 | 55934 | 0.74 | 0.92 | 0.0008 | 0.0003 | 0.3285 | 71 |
| Rpain | 69723 | 0.55 | 1.03 | 0.0001 | 0.0003 | 0.8024 | 107 |
| Rpp38 | n/a | 0.52 | 1.05 | 0.0010 | 0.0001 | 0.7117 | 111 |
| Rtn3 | 20168 | 1.60 | 1.00 | 0.0017 | 0.0020 | 0.9698 | 99 |
| Rtp3 | 235636 | 0.46 | 0.86 | 0.0001 | 0.0052 | 0.2295 | 74 |
| S100a10 | 20194 | 1.49 | 1.00 | 0.0010 | 0.0026 | 0.9713 | 101 |
| Saa2 | 20209 | 0.25 | 1.30 | 0.0034 | 0.0000 | 0.2739 | 139 |
| Saa4 | 20211 | 0.51 | 1.11 | 0.0053 | 0.0026 | 0.5097 | 123 |
| Sdc1 | 20969 | 1.75 | 1.76 | 0.0004 | 0.9384 | 0.0018 | -1 |
| Sema4a | 20351 | 0.60 | 0.61 | 0.0003 | 0.8042 | 0.0033 | 2 |
| Sephs2 | 20768 | 1.48 | 1.29 | 0.0011 | 0.1111 | 0.0257 | 41 |
| Sept9 | 53860 | 1.80 | 1.02 | 0.0083 | 0.0175 | 0.8471 | 97 |
| Sergef | 27414 | 0.62 | 0.71 | 0.0007 | 0.3173 | 0.0177 | 23 |
| Serpina10 | 217847 | 0.51 | 0.79 | 0.0000 | 0.0000 | 0.0212 | 58 |
| Serpina3k | 20714 | 1.56 | 1.36 | 0.0028 | 0.1531 | 0.0230 | 35 |
| Serpinh1 | 12406 | 2.02 | 1.20 | 0.0041 | 0.0347 | 0.2826 | 81 |
| Sesn1 | 140742 | 2.17 | 1.22 | 0.0005 | 0.0049 | 0.0318 | 81 |
| Setd1a | 233904 | 1.47 | 1.33 | 0.0028 | 0.3574 | 0.0458 | 31 |
| Setdb2 | 239122 | 3.00 | 1.78 | 0.0033 | 0.0788 | 0.0113 | 61 |
| Sf3a3 | 75062 | 0.72 | 0.88 | 0.0001 | 0.0029 | 0.1348 | 58 |
| Sfrs1 | 110809 | 0.66 | 0.96 | 0.0000 | 0.0022 | 0.6800 | 89 |
| Sfrs3 | 20383 | 0.62 | 0.99 | 0.0002 | 0.0005 | 0.9612 | 99 |
| Sfrs4 | 57317 | 1.52 | 1.48 | 0.0013 | 0.7423 | 0.0166 | 8 |
| Sfrs7 | 225027 | 0.39 | 0.58 | 0.0000 | 0.0100 | 0.0007 | 30 |
| Sgk2 | 27219 | 1.76 | 1.31 | 0.0001 | 0.0601 | 0.1874 | 59 |
| Sgms2 | 74442 | 2.00 | 0.60 | 0.0015 | 0.0001 | 0.0522 | 140 |
| Sgpl1 | 20397 | 1.62 | 1.19 | 0.0008 | 0.0138 | 0.0839 | 70 |
| Sh3bp5l | 79566 | 0.45 | 0.67 | 0.0000 | 0.0021 | 0.0016 | 39 |
| Sh3yl1 | 24057 | 0.59 | 0.57 | 0.0014 | 0.7464 | 0.0067 | -4 |
| Siah2 | 20439 | 0.62 | 0.72 | 0.0019 | 0.2606 | 0.0503 | 27 |
| Sidt2 | 214597 | 1.44 | 1.45 | 0.0041 | 0.9524 | 0.0374 | -3 |
| Skap2 | 54353 | 0.60 | 0.94 | 0.0000 | 0.0000 | 0.4750 | 86 |
| Slc15a4 | 100561 | 2.00 | 1.75 | 0.0000 | 0.2414 | 0.0011 | 25 |
| Slc20a1 | 20515 | 2.09 | 1.56 | 0.0058 | 0.1412 | 0.0173 | 48 |
| Slc25a15 | 18408 | 1.55 | 2.26 | 0.0009 | 0.0001 | 0.0000 | -128 |
| Slc25a33 | 70556 | 1.43 | 1.30 | 0.0013 | 0.2662 | 0.0047 | 31 |
| Slc25a42 | 73095 | 1.60 | 1.04 | 0.0080 | 0.0442 | 0.8024 | 93 |
| Slc25a45 | 107375 | 0.68 | 0.66 | 0.0017 | 0.7774 | 0.0024 | -6 |
| Slc30a1 | 22782 | 1.57 | 0.88 | 0.0000 | 0.0000 | 0.3395 | 122 |
| Slc30a5 | 69048 | 0.60 | 1.21 | 0.0001 | 0.0000 | 0.0879 | 152 |
| Slc37a1 | 224674 | 0.15 | 0.86 | 0.0002 | 0.0001 | 0.4805 | 84 |
| Slc41a2 | 338365 | 0.35 | 1.82 | 0.0004 | 0.0003 | 0.0503 | 225 |
| Slc6a6 | 21366 | 1.45 | 1.07 | 0.0012 | 0.0010 | 0.5951 | 85 |
| Slc6a9 | 14664 | 0.60 | 0.88 | 0.0019 | 0.0388 | 0.1274 | 70 |
| Slc9a1 | 20544 | 0.63 | 0.60 | 0.0017 | 0.6373 | 0.0053 | -7 |
| Slco2a1 | 24059 | 1.50 | 1.16 | 0.0069 | 0.0516 | 0.2262 | 67 |
| Smarcad1 | 13990 | 0.60 | 0.94 | 0.0002 | 0.0024 | 0.3329 | 85 |
| Smarcc2 | 68094 | 1.65 | 1.09 | 0.0030 | 0.0263 | 0.6210 | 86 |
| Smarcd2 | 83796 | 0.70 | 0.57 | 0.0012 | 0.0127 | 0.0014 | -43 |
| Smek2 | 104570 | 0.64 | 0.87 | 0.0006 | 0.0003 | 0.2223 | 64 |
| Snip1 | 76793 | 0.55 | 1.02 | 0.0000 | 0.0001 | 0.8433 | 104 |
| Snupn | 66069 | 0.64 | 0.64 | 0.0019 | 0.9827 | 0.0050 | 0 |
| Snx10 | 71982 | 0.43 | 1.43 | 0.0002 | 0.0000 | 0.0250 | 176 |
| Snx11 | 74479 | 0.52 | 0.81 | 0.0002 | 0.0013 | 0.0687 | 61 |
| Snx18 | 170625 | 1.47 | 1.11 | 0.0001 | 0.0019 | 0.2028 | 78 |
| Snx24 | 69226 | 1.43 | 1.12 | 0.0000 | 0.0006 | 0.0207 | 72 |
| Soat2 | 223920 | 0.57 | 0.82 | 0.0003 | 0.0077 | 0.1422 | 59 |
| Spnb3 | 20743 | 1.41 | 0.97 | 0.0005 | 0.0024 | 0.7884 | 108 |
| Sppl3 | 74585 | 0.72 | 0.74 | 0.0026 | 0.6448 | 0.0188 | 8 |
| Spsb1 | 74646 | 2.16 | 1.98 | 0.0013 | 0.7541 | 0.1405 | 16 |
| Srfbp1 | 67222 | 0.30 | 0.74 | 0.0001 | 0.0001 | 0.0847 | 63 |
| Stat1 | 20846 | 0.59 | 0.84 | 0.0000 | 0.0019 | 0.0612 | 61 |
| Stat2 | 20847 | 0.51 | 1.14 | 0.0000 | 0.0000 | 0.1704 | 128 |
| Stat3 | 20848 | 0.52 | 1.01 | 0.0000 | 0.0000 | 0.9316 | 102 |
| Stip1 | 20867 | 1.46 | 1.38 | 0.0025 | 0.5266 | 0.0122 | 18 |
| Stk11 | 20869 | 1.36 | 1.14 | 0.0037 | 0.0524 | 0.1824 | 60 |
| Stk24 | n/a | 1.40 | 1.06 | 0.0001 | 0.0008 | 0.3371 | 85 |
| Stk38l | 232533 | 0.58 | 0.62 | 0.0001 | 0.5889 | 0.0023 | 10 |
| Stx18 | 71116 | 0.66 | 1.12 | 0.0006 | 0.0226 | 0.5811 | 135 |
| Stx5a | 56389 | 0.62 | 0.87 | 0.0000 | 0.0006 | 0.1051 | 65 |
| Syvn1 | 74126 | 0.72 | 1.01 | 0.0002 | 0.0148 | 0.9573 | 102 |
| Tapt1 | 231225 | 0.64 | 0.86 | 0.0000 | 0.0005 | 0.0899 | 62 |
| Tatdn2 | 381801 | 1.66 | 1.69 | 0.0016 | 0.8397 | 0.0059 | -5 |
| Tbc1d13 | 70296 | 1.50 | 0.89 | 0.0056 | 0.0017 | 0.5081 | 123 |
| Tbp | 21374 | 0.56 | 0.98 | 0.0000 | 0.0000 | 0.7221 | 96 |
| Tbx3 | 21386 | 0.37 | 0.59 | 0.0001 | 0.0067 | 0.0153 | 35 |
| Tfb1m | 224481 | 0.61 | 0.56 | 0.0024 | 0.4657 | 0.0072 | -12 |
| Tfrc | 22042 | 1.96 | 2.98 | 0.0004 | 0.0348 | 0.0024 | -106 |
| Tgm1 | 21816 | 0.51 | 0.90 | 0.0002 | 0.0029 | 0.4478 | 79 |
| Tha1 | 71776 | 0.45 | 0.61 | 0.0001 | 0.0012 | 0.0083 | 29 |
| Tlcd1 | 68385 | 0.65 | 0.62 | 0.0002 | 0.6664 | 0.0018 | -9 |
| Tmem185b | 226351 | 0.54 | 0.66 | 0.0001 | 0.0277 | 0.0052 | 26 |
| Tmem186 | 66690 | 0.33 | 0.68 | 0.0000 | 0.0004 | 0.0141 | 52 |
| Tmem199 | 195040 | 0.54 | 0.68 | 0.0001 | 0.0341 | 0.0187 | 31 |
| Tmem39a | 67846 | 0.55 | 1.16 | 0.0034 | 0.0014 | 0.4897 | 135 |
| Tmprss2 | 50528 | 2.03 | 1.86 | 0.0009 | 0.5180 | 0.0025 | 16 |
| Tmprss6 | n/a | 1.49 | 1.22 | 0.0013 | 0.0110 | 0.1192 | 55 |
| Tnfrsf12a | 27279 | 1.89 | 2.00 | 0.0071 | 0.7570 | 0.0099 | -13 |
| Tob1 | 22057 | 2.13 | 1.19 | 0.0117 | 0.0696 | 0.4825 | 83 |
| Tpm4 | 326618 | 1.53 | 1.34 | 0.0043 | 0.4674 | 0.2051 | 36 |
| Trak1 | 67095 | 1.37 | 1.40 | 0.0035 | 0.8453 | 0.0323 | -9 |
| Trfp | 56771 | 0.41 | 0.65 | 0.0000 | 0.0017 | 0.0197 | 41 |
| Trim39 | 79263 | 0.57 | 1.19 | 0.0000 | 0.0002 | 0.2421 | 143 |
| Trim56 | 384309 | 0.58 | 0.80 | 0.0003 | 0.0271 | 0.0302 | 52 |
| Trp53bp2 | 209456 | 1.95 | 1.72 | 0.0001 | 0.2248 | 0.0002 | 25 |
| Trp53inp1 | 60599 | 3.43 | 2.71 | 0.0002 | 0.1753 | 0.0002 | 30 |
| Tut1 | 70044 | 0.46 | 0.81 | 0.0000 | 0.0000 | 0.0253 | 64 |
| Ube2m | 22192 | 1.49 | 1.54 | 0.0036 | 0.7838 | 0.0093 | -9 |
| Ugt2b34 | 100727 | 1.42 | 1.23 | 0.0063 | 0.2413 | 0.0739 | 45 |
| Upf2 | 326622 | 0.58 | 0.81 | 0.0001 | 0.0003 | 0.0910 | 54 |
| Uvrag | 78610 | 1.86 | 1.27 | 0.0000 | 0.0009 | 0.1611 | 68 |
| Vmo1 | 327956 | 0.65 | 0.74 | 0.0008 | 0.4172 | 0.0165 | 24 |
| Vps37b | 330192 | 1.77 | 1.11 | 0.0010 | 0.0064 | 0.1440 | 85 |
| Vrk3 | 101568 | 0.62 | 0.75 | 0.0001 | 0.0036 | 0.0167 | 33 |
| Wdr20a | 69641 | 0.63 | 0.98 | 0.0005 | 0.0033 | 0.8368 | 95 |
| Whsc2 | 24116 | 0.56 | 0.80 | 0.0001 | 0.0090 | 0.0995 | 55 |
| Wwc2 | 52357 | 0.67 | 0.83 | 0.0003 | 0.0376 | 0.0103 | 48 |
| X99384 | 27355 | 1.51 | 1.10 | 0.0000 | 0.0013 | 0.2257 | 79 |
| Xbp1 | 22433 | 0.43 | 0.75 | 0.0000 | 0.0003 | 0.0455 | 57 |
| Ythdf1 | 228994 | 0.72 | 1.02 | 0.0007 | 0.0004 | 0.8258 | 107 |
| Zbtb24 | 268294 | 0.63 | 0.77 | 0.0014 | 0.1877 | 0.0164 | 36 |
| Zc3h7a | 106205 | 0.70 | 1.24 | 0.0002 | 0.0000 | 0.0431 | 181 |
| Zfp207 | 22680 | 0.65 | 1.04 | 0.0000 | 0.0026 | 0.7115 | 112 |
| Zfp263 | 74120 | 0.74 | 0.91 | 0.0001 | 0.0019 | 0.1746 | 66 |
| Zfp282 | 101095 | 0.50 | 0.73 | 0.0004 | 0.0166 | 0.0250 | 45 |
| Zfp36 | 22695 | 2.24 | 1.87 | 0.0009 | 0.2900 | 0.0105 | 30 |
| Zfp46 | 22704 | 0.46 | 0.75 | 0.0001 | 0.0009 | 0.0507 | 53 |
| Zfp574 | 232976 | 0.67 | 0.71 | 0.0028 | 0.4405 | 0.0219 | 12 |
| Zfp597 | 71063 | 0.55 | 0.94 | 0.0001 | 0.0012 | 0.5281 | 86 |
| Zfp60 | 22718 | 0.24 | 0.85 | 0.0000 | 0.0000 | 0.2449 | 80 |
| Zfp672 | 319475 | 0.52 | 0.72 | 0.0003 | 0.0027 | 0.0378 | 42 |
| Zfp825 | 235956 | 0.58 | 0.85 | 0.0000 | 0.0032 | 0.1331 | 65 |
| Zfp94 | 22756 | 0.18 | 0.44 | 0.0000 | 0.0308 | 0.0066 | 32 |
| Zfx | 22764 | 0.65 | 0.94 | 0.0001 | 0.0005 | 0.2258 | 82 |
| Zhx3 | 320799 | 1.53 | 1.20 | 0.0001 | 0.0246 | 0.0796 | 63 |
| Zranb1 | 360216 | 1.43 | 1.51 | 0.0001 | 0.5130 | 0.0058 | -19 |
| Zswim4 | 212168 | 1.96 | 1.77 | 0.0001 | 0.2252 | 0.0002 | 21 |
