# Supplementary material for: Acute Overactive Endocannabinoid Signaling Induces Glucose Intolerance, Hepatic Steatosis, and Novel Cannabinoid Receptor 1 Responsive Genes
Source: PLoS One. 2011 Nov 4;6(11):e26415. doi: 10.1371/journal.pone.0026415 (PMC3208546; doi:10.1371/journal.pone.0026415)
Supplement: Table S4 — Panther Biological Process Analysis Biological processes found to be significantly altered by Panther analysis. (DOCX) [file pone.0026415.s006.docx]

**Supplemental Table 4: Panther Biological Process Analysis**

| **Biological Process** | **number** | **±** | **P value** |
| --- | --- | --- | --- |
| **DMSO vs. IDFP** |  |  |  |
| Cell adhesion | 175 | + | 0.00003 |
| Biological process unclassified | 2524 | - | 0.00005 |
| Cell structure and motility | 389 | + | 0.00181 |
| Homeostasis | 80 | + | 0.00315 |
| Lipid, fatty acid and steroid metabolism | 401 | + | 0.00354 |
| Extracellular matrix protein-mediated signaling | 30 | + | 0.00551 |
| Sulfur metabolism | 42 | + | 0.01200 |
| Cell communication | 318 | + | 0.01750 |
| Fatty acid metabolism | 109 | + | 0.04710 |
| Growth factor homeostasis | 7 | + | 0.04810 |
| **IDFP vs. IDFP/Am251** |  |  |  |
| rRNA metabolism | 45 | + | 0.00281 |
| Lipid, fatty acid and steriod metabolism | 401 | + | 0.00471 |
