# Supplementary material for: Acute Overactive Endocannabinoid Signaling Induces Glucose Intolerance, Hepatic Steatosis, and Novel Cannabinoid Receptor 1 Responsive Genes
Source: PLoS One. 2011 Nov 4;6(11):e26415. doi: 10.1371/journal.pone.0026415 (PMC3208546; doi:10.1371/journal.pone.0026415)
Supplement: Table S5 — FuncAssociate 2.0 Analysis of Genes Decreased by IDFP in a CB1 Dependent Manner Summary of FuncAssocaite analysis of genes (n = 168) downregulated by IDFP and reversed by Am251(>50%). (DOCX) [file pone.0026415.s007.docx]

**Supplemental Table 5: FuncAssociate 2.0 Analysis of Genes Decreased by IDFP in a CB1 Dependent Manner**

| **Attribute name** | **Attribute ID** | **N** | **X** | **LOD** | **P_adj** |
| --- | --- | --- | --- | --- | --- |
| acute-phase response | GO:0006953 | 5 | 28 | 1.37 | 0.008 |
| aminoacyl-tRNA ligase activity | GO:0004812 | 8 | 45 | 1.37 | <0.001 |
| ligase activity, forming carbon-oxygen bonds | GO:0016875 | 8 | 45 | 1.37 | <0.001 |
| ligase activity, forming aminoacyl-tRNA and related compounds | GO:0016876 | 8 | 45 | 1.37 | <0.001 |
| tRNA aminoacylation for protein translation | GO:0006418 | 7 | 42 | 1.33 | <0.001 |
| amino acid activation | GO:0043038 | 7 | 42 | 1.33 | <0.001 |
| tRNA aminoacylation | GO:0043039 | 7 | 42 | 1.33 | <0.001 |
| acute inflammatory response | GO:0002526 | 6 | 40 | 1.28 | 0.001 |
| tRNA metabolic process | GO:0006399 | 11 | 107 | 1.09 | <0.001 |
| ncRNA metabolic process | GO:0034660 | 11 | 180 | 0.85 | 0.001 |
| translation | GO:0006412 | 12 | 252 | 0.73 | 0.008 |
| RNA metabolic process | GO:0016070 | 20 | 544 | 0.62 | 0 |
