# Supplementary material for: Acute Overactive Endocannabinoid Signaling Induces Glucose Intolerance, Hepatic Steatosis, and Novel Cannabinoid Receptor 1 Responsive Genes
Source: PLoS One. 2011 Nov 4;6(11):e26415. doi: 10.1371/journal.pone.0026415 (PMC3208546; doi:10.1371/journal.pone.0026415)
Supplement: Table S6 — Expression of Genes involved in Stat3 signaling and Lipid Metabolism in WT and CB1 −/− Mice Wild-type and CB1 −/− mice were treated with DMSO or IDFP (10 mg/kg, ip, 4 h). Groups not sharing a common superscript letter are significantly different (p<0.05). n = 5. (DOCX) [file pone.0026415.s008.docx]

**Supplemental Table 6: Expression of Genes involved in Stat3 signaling and Lipid Metabolism in WT and CB1 -/- Mice**

| Gene | WT DMSO | WT IDFP | CB1 -/- DMSO | CB1 -/- IDFP |
| --- | --- | --- | --- | --- |
| **Stat3 Signaling** |  |  |  |  |
| stat3 | 1.00 ± 0.15 | 0.43 ± 0.02 | 0.96 ± 0.29 | 0.84 ± 0.14 |
| lbp | 1.00 ± 0.12 | 0.78 ± 0.13 | 1.00 ± 0.28 | 1.11 ± 0.13 |
| apcs | 1.00 ± 0.19 | 0.61 ± 0.16 | 0.69 ± 0.27 | 0.64 ± 0.11 |
| **Lipid Metabolism** |  |  |  |  |
| acsl1 | 1.00 ± 0.09^ab^ | 1.61 ± 0.34^a^ | 1.04 ± 0.16^ab^ | 0.73 ± 0.10^b^ |
| insig1 | 1.00 ± 0.16^a^ | 2.35 ± 0.41^b^ | 0.81 ± 0.19^a^ | 0.81 ± 0.16^a^ |
| pgc1b | 1.00 ± 0.14^a^ | 4.28 ± 0.25^b^ | 1.28 ± 0.29^a^ | 2.34 ± 0.37^c^ |
| ldlr | 1.00 ± 0.23^a^ | 1.50 ± 0.32^b^ | 1.05 ± 0.49^a^ | 0.81 ± 0.18^a^ |
| lpin2 | 1.00 ± 0.27 | 1.72 ± 0.25 | 1.03 ± 0.10 | 1.64 ± 0.24 |
| hmgcr | 1.00 ± 0.04 | 2.17 ± 0.56 | 1.09 ± .026 | 1.21 ± 0.45 |

Groups not sharing a common superscript letter are significantly different (p<0.05).
