# Supplementary material for: Acute Overactive Endocannabinoid Signaling Induces Glucose Intolerance, Hepatic Steatosis, and Novel Cannabinoid Receptor 1 Responsive Genes
Source: PLoS One. 2011 Nov 4;6(11):e26415. doi: 10.1371/journal.pone.0026415 (PMC3208546; doi:10.1371/journal.pone.0026415)
Supplement: Table S7 — Known off target effects of IDFP Enzymes known to be altered by IDFP. (DOCX) [file pone.0026415.s009.docx]

**Supplemental Table 7: Known off target effects of IDFP**

| **Off-targets of IDFP** | **Physiological role** |
| --- | --- |
| KIAA1363 | Hydrolyzes acetyl monoalkyglycerol ether of the platelet-activating factor de novo biosynthetic pathway [1] |
| Neuropathy target esterase | Blocking NTE results in delayed neurotoxicity and peripheral demyelination[[1](#_ENREF_1)] |
| Neuropathy target esterase related esterase | unknown |
| Abhydrolase domain containing 6 | unknown |
| Acylamino acid releasing protein | Not completely understood--implicated in hydrolysis of terminal acetylated amino acids from peptides[[2](#_ENREF_2)] |
| Abhydrolase domain containing 3 | unknown |
| CE-N | unknown |
| Hormone sensitive lipase | Involved in diacylglycerol metabolism[[3](#_ENREF_3)] |

**References**

**1. Casida JE, Nomura DK, Vose SC, Fujioka K (2008) Organophosphate-sensitive lipases modulate brain lysophospholipids, ether lipids and endocannabinoids. Chem Biol Interact 175: 355-364.**

**2. Perrier J, Durand A, Giardina T, Puigserver A (2005) Catabolism of intracellular N-terminal acetylated proteins: involvement of acylpeptide hydrolase and acylase. Biochimie 87: 673-685.**

**3. Haemmerle G, Zimmermann R, Hayn M, Theussl C, Waeg G, et al. (2002) Hormone-sensitive lipase deficiency in mice causes diglyceride accumulation in adipose tissue, muscle, and testis. J Biol Chem 277: 4806-4815.**
